# On Recovery of a Non-Negative Relaxation Spectrum Model from the Stress Relaxation Test Data

**DOI:** 10.3390/polym15163464

**Published:** 2023-08-18

**Authors:** Anna Stankiewicz, Monika Bojanowska, Paweł Drozd

**Affiliations:** 1 Department of Technology Fundamentals, Faculty of Production Engineering, University of Life Sciences in Lublin, 20-612 Lublin, Poland; pawel.drozd@up.lublin.pl; 2 Department of Chemistry, Faculty of Food Science and Biotechnology, University of Life Sciences in Lublin, 20-950 Lublin, Poland; monika.bojanowska@up.lublin.pl

**Keywords:** relaxation spectrum, linear relaxation modulus, non-negative model, identification algorithm, least-squares identification, smoothing constraint, dual optimization problem

## Abstract

The relaxation spectra, from which other material functions used to describe mechanical properties of materials can be uniquely determined, are important for modeling the rheological properties of polymers used in chemistry, food technology, medicine, cosmetics, and many other industries. The spectrum, being not directly accessible by measurement, is recovered from relaxation stress or oscillatory shear data. Only a few models and identification methods take into account the non-negativity of the real spectra. In this paper, the problem of recovery of non-negative definite relaxation spectra from discrete-time noise-corrupted measurements of relaxation modulus obtained in the stress relaxation test is considered. A new hierarchical identification scheme is developed, being applicable both for relaxation time and frequency spectra. Finite-dimensional parametric classes of models are assumed for the relaxation spectra, described by a finite series of power-exponential and square-exponential basis functions. The related models of relaxation modulus are given by compact analytical formula, described by the products of power of time and the modified Bessel functions of the second kind for the time spectrum, and by recurrence formulas based on products of power of time and complementary error functions for frequency spectrum. The basis functions are non-negative. In result, the identification task was reduced to a finite-dimensional linear-quadratic problem with non-negative unknown model parameters. To stabilize the solution, an additional smoothing constraint is introduced. Dual approach was used to solve the stated optimal identification task resulting in the hierarchical two-stage identification scheme. In the first stage, dual problem is solved in two levels and the vector of non-negative model parameters is computed to provide the best fit of the relaxation modulus to experiment data. Next, in second stage, the optimal non-negative spectrum model is determined. A complete scheme of the hierarchical computations is outlined; it can be easily implemented in available computing environments. The model smoothness is analytically studied, and the applicability ranges are numerically examined. The numerical studies have proved that using developed models and algorithm, it is possible to determine non-negative definite unimodal and bimodal relaxation spectra for a wide class of polymers. However, the examples also demonstrated that if the basis functions are non-negative and the model is properly selected for a given type of the real spectrum (unimodal, multimodal), the optimal model determined without non-negativity constraint can be non-negative in the dominant range of its arguments, especially in the wide neighborhood of the spectrum peaks.

## 1. Introduction

The viscoelastic relaxation spectrum provides deep insights into the complex behavior of polymers [1,2,3]. The spectrum is not directly measurable and must be recovered from oscillatory shear or relaxation stress data [1,3]. During the last five decades, a number of models and methods have been proposed for the recovery of the relaxation spectrum of a viscoelastic material from oscillatory shear data. The contributions by Baumgaertel and Winter [4], Honerkamp and Weese [5], Malkin [6], Malkin et al. [7], Stadler and Bailly [8], Davis and Goulding [9], Davis et al. [10], and Cho [11] are most frequently cited, as they laid the foundations for several parallel directions of research on the identification of discrete and continuous relaxation spectra based on dynamic modulus data.

Far fewer methods have been proposed for spectrum determination from time-measurements of the relaxation modulus collected in the stress relaxation test, where time-dependent shear stress is studied for the step increase in the strain. Additionally, some of them only address specific materials. A concise discussion of these works, among which three directions of research can be distinguished, is given in [12]. The three indicated classes of approaches are: (1) differential models and algorithms based on applying the Post–Widder formula [13] of the inverse Laplace transform to designate the relaxation spectrum models proposed in the papers of Alfrey and Doty [14], Ter Haar [15], Bažant and Yunping [16], Goangseup and Bažant [17]; (2) the models derived directly from the known pairs of Laplace transforms proposed by Macey [18], Sips [19,20] and Yamamoto [21] and (3) models based on the expansion of an unknown spectrum into a series of basis functions forming a complete basis in the space of real-valued square-integrable functions developed by Stankiewicz [12,22,23] and Stankiewicz and Gołacki [24]. Some articles are also discussed below.

The relaxation spectra of real materials are non-negative for any relaxation time and any relaxation frequency. However, most of the known models and identification algorithms do not take into account this non-negativity property. Therefore, the resulting spectrum model may take a negative value for some relaxation times or frequencies. The exceptions are those methods that use the spectrum approximation by non-negative definite simple functions, represented by the Macey [18] exponential-hyperbolic model of the spectrum, the Sips [19,20] model given by difference of two exponential functions, next augmented by Yamamoto [21] to consider long-term modulus. However, resulting spectrum models are positive for all arguments; the scope of their effective applicability is limited due to rather narrow classes of models. The Alfrey and Doty [14] simple differential model, the Ter Harr [15] approximation of the spectrum of relaxation frequencies by the modulus multiplied by the time inverse of the relaxation frequency and other methods using the Post-Widder inversion formula to designate the relaxation spectrum model, as Bažant and Yunping [16] and Goangseup and Bažant [17] two-stage approaches of approximating the stress data by multiple differentiable models of relaxation modulus and next, applying the Post-Widder formula to compute the related spectrum model, guarantee the positive definiteness of the recovered relaxation spectrum whenever the relaxation modulus is a completely monotonic function [25]. Thus, the ranges of their applicability are restricted, also due to the necessity of multiple differentiation of the noise corrupted measurement data. A wider range of applicability has been obtained by Stankiewicz [22] for the non-negative model based on the expanding of an unknown spectrum of relaxation frequencies into a series of basis power-exponential functions. However, article [22] was based on such a definition of the relaxation spectrum, which is not often used in the literature. 

Therefore, the goal of the present paper was to formulate and solve the problem of determination of the non-negative definite model of the relaxation spectrum based on discrete-time measurements of the relaxation modulus obtained in the relaxation test. 

It was assumed that the approach’s proposed and developed identification scheme will be applicable to determine both the relaxation time and frequency spectra. The approximation of the continuous spectrum by finite series of non-negative basis functions was applied. For modeling, the relaxation time spectrum model introduced in [12] was used, while for the spectrum of relaxation frequencies, the basis functions described by the product of power of time and square exponential functions were applied. The components of the relaxation modulus model are given by compact recurrence formulas expressed in terms of the products of power of time, exponential, and complementary error function. Both classes of models depend on some time-scale factors. The main properties of the basis functions of relaxation spectrum and modulus models have been studied; positive definiteness, monotonicity, and asymptotic properties have been examined. Ranges of applicability for different scale-time factors were determined. 

A quadratic identification index, which refers to the measured relaxation modulus, was adopted. In result, the original continuous, infinite-dimensional, task of determining the best non-negative definite function, was reduced to a static, finite-dimensional, linear-quadratic optimization problem with a non-negativity constraint imposed on the vector of model parameters. The smoothing constraint for the vector of model parameters was introduced to achieve the well-posed optimization task. It is proved that the smoothness of the optimal parameters vector implies smoothness of the fluctuations of the relaxation spectrum model. Direct formula, upper, and lower bounds for the square integral norm of the smoothed spectrum model are derived in terms of the smoothing parameter.

Next, the dual approach was applied to solve the stated linear-quadratic constrained optimization task, resulting in the two-stage hierarchical identification scheme. The existence of the dual problem solution was proved. A parametric approach of successive optimization was applied to solve the dual maximization task. The optimality condition for the partial dual task was derived in the form of a simple algebraic equation. A hierarchical two-stage identification scheme was proposed. The maximization dual task was solved in two levels of the first stage, while the optimal model was determined in the second stage of the scheme. The numerical realization based on applying the singular value decomposition technique is discussed. A complete computational algorithm is outlined. The identification scheme can be easily implemented in commonly used computing environments. The numerical studies were conducted for examples of Gauss-like spectra. Both unimodal spectrum, typical for many polymers, e.g., example polymers used in food technology [26], and bimodal spectra equally often used to describe rheological properties of various polymers [27], e.g., polyacrylamide gels [28] and polymers used in food technology [29,30,31], were modeled. The examples and other numerical studies have proved that using the algorithm, it is possible to determine non-negative definite unimodal and bimodal relaxation spectra for a wide class of polymers. However, the examples also show that, in practice, the non-negative models of the relaxation spectra or models non-negative for almost all arguments can be obtained also using the classical approach, without the additional constraint of the non-negativity of the model parameters, if the basis functions of the relaxation spectra models are non-negatively defined.

In Appendix A, the proofs and derivations of some mathematical formulas are given. Some tables have been moved to Appendix B, to increase the clarity of the article.

## 2. Materials and Methods

### 2.1. Spectrum of Relaxation

The uniaxial, nonaging, and isothermal stress–strain equation for a linear viscoelastic material can be represented by a Boltzmann superposition integral [3]:

$$\sigma \left(t\right)=\int_{-\infty }^{t}G\left(t-u\right)\dot{\varepsilon }\left(u\right)du,$$

where σ(t) and ε(t) denote the stress and strain at the time t and G(t) is the linear relaxation modulus. Modulus G(t) is given by [1,3,12]:

(1) $$G\left(t\right)=\int_{0}^{\infty }\frac{ℋ\left(\tau \right)}{\tau }e^{-t/\tau }d\tau ,$$

or, equivalently, by [1,3]

(2) $$G\left(t\right)=\int_{0}^{\infty }\frac{H\left(v\right)}{v}e^{-tv}dv,$$

where ℋ(τ) and H(v) characterize the distributions of relaxation times τ and relaxation frequencies v, respectively. The continuous relaxation spectra ℋ(τ) and H(v), related by $H\left(v\right)=ℋ\left(\frac{1}{v}\right)$, are generalizations of the discrete Maxwell spectrum [1,3] to a continuous function of the relaxation times τ and frequencies v. Although other definitions of the relaxation spectrum are used in the literature; for example, in [6,22,24,32,33], the definitions introduced by Equations (1) and (2) dominate. 

The problem of relaxation spectrum recovery from measurement data, i.e., the problem of solving system of Fredholm integral equations of the first kind (1) or (2), is known to be ill-posed in the Hadamard sense [34], i.e., small changes in measured relaxation modulus can lead to arbitrarily large changes in the determined relaxation spectrum. In remedy, some reduction in the set of admissible solutions can be used. Spectra of relaxation times and frequencies will be modeled by non-negative definite finite series of non-negative basis functions. 

### 2.2. Model of Relaxation Time Spectrum

Assume that $ℋ\left(\tau \right)\in L^{2}\left(0,\infty \right)$, where $L^{2}(0,\infty )$ is the space of real-valued square-integrable functions on the interval (0,∞). The set of the linearly independent functions $\left\{e^{-\alpha \tau },\tau e^{-\alpha \tau },\tau^{2}e^{-\alpha \tau },\ldots \right\}$ form a basis of the space $L^{2}(0,\infty )$ [35]; here α is positive time-scaling factor. Since the maxima of these basis functions grows rapidly with k, in [12] the scaled basis functions:

(3) $$h_{k}\left(\tau ,\alpha \right)={\left(\frac{\alpha \tau }{k}\right)}^{k}e^{-\alpha \tau +k},\;k=0,1,\ldots ,$$

with the first function

(4) $$h_{0}\left(\tau ,\alpha \right)=e^{-\alpha \tau },$$

are assumed to approximate the relaxation time spectrum ℋ(τ) by the model [12]:

(5) $${ℋ}_{K}\left(\tau ,\alpha \right)=\sum_{k=0}^{K-1}g_{k}h_{k}\left(\tau ,\alpha \right),$$

where the lower index is the number of model summands. Function $h_{0}\left(\tau ,\alpha \right)$ (4) is defined for computational purposes only, since $0^{0}=1$ [36], i.e., following [12], the general Formula (3) can be applied in further analysis also for k=0.

Then, according to Equation (1), the respective model of the relaxation modulus is described by:

(6) $$G_{K}\left(t,\alpha \right)=\int_{0}^{\infty }\frac{{ℋ}_{K}\left(\tau ,\alpha \right)}{\tau }e^{-t/\tau }d\tau =\sum_{k=0}^{K-1}g_{k}\varphi_{k}\left(t,\alpha \right),$$

where the basis functions for the spectrum model (6) are given by compact analytical formula specified by the following theorem proved in [12].

**Theorem** **1** **[12].** *Let* α>0, k≥0 *and* t>0*. Then the basis functions* $\varphi_{k}\left(t,\alpha \right)$ *are given by:*

(7) $$\varphi_{k}\left(t,\alpha \right)=\int_{0}^{\infty }\frac{h_{k}\left(\tau ,\alpha \right)}{\tau }e^{-t/\tau }d\tau =2e^{k}{\left(\frac{\sqrt{\alpha t}}{k}\right)}^{k}K_{k}\left(2\sqrt{\alpha t}\right)\;,$$

*where* $K_{k}\left(x\right)$ *is the modified Bessel function of the second kind [37] of integer order k*.

**Figure 1:**
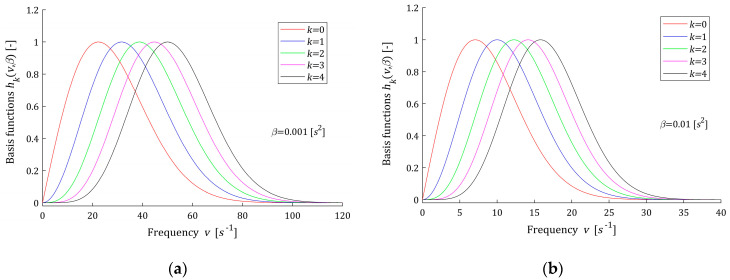
Basis functions $\phi_{k}\left(t,\beta \right)$ (13) of the relaxation spectrum model $H_{K}\left(v,\beta \right)$ (10) for time-scaling factors: (**a**) $\beta =0.001\;\left[s^{2}\right]$ and (**b**) $\beta =0.01\;\left[s^{2}\right],\;k=0,1,2,3,4$.

**Figure 2:**
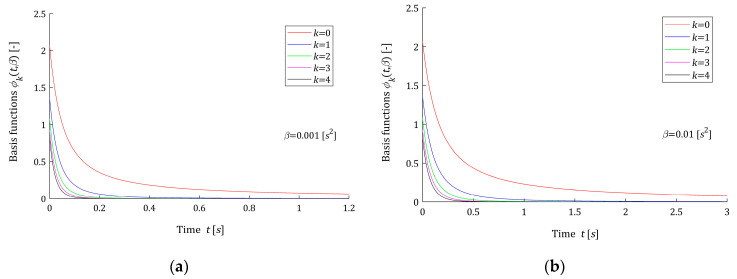
Basis functions $\phi_{k}\left(t,\beta \right)$ (14)–(16) of the relaxation modulus model $G_{K}\left(t,\beta \right)$ (12) for time-scaling factors: (**a**) $\beta =0.001\;\left[s^{2}\right]$ and (**b**) $\beta =0.01\;\left[s^{2}\right],\;k=0,1,2,3,4$.

The courses of the dimensionless basis functions $h_{k}\left(\tau ,\alpha \right)$ (3) and $\varphi_{k}\left(t,\alpha \right)$ (7) are shown and discussed in [12] (Figures 1 and 2). In [12] the properties of the basis functions $h_{k}\left(\tau ,\alpha \right)$ and $\varphi_{k}\left(t,\alpha \right)$ were examined, their positive definiteness [12] (Section 2.2.1) and asymptotic convergence $\varphi_{k}\left(t,\alpha \right)\to 0$ as t→∞ [12] (Section 2.2.2) were proved. Their monotonicity was also examined [12] (Section 2.2.4) and ranges of applicability were determined for a wide range of the time-scale factor α [12] (Section 2.2.5). 

### 2.3. Model of Relaxation Frequency Spectrum

Assume that the spectrum introduced in Equation (2) is such that $H\left(v\right)\in L^{2}(0,\infty )$. The set of the linearly independent functions $\left\{e^{-\beta v^{2}},ve^{-\beta v^{2}},v^{2}e^{-\beta v^{2}},\ldots \right\}$ form a basis of the space $L^{2}(0,\infty )$ [38]; here β is a positive time-scaling factor; more precisely, a square of the time-scale factor $\sqrt{\beta }$ expressed in seconds. 

Since for any fixed β the maximum:

$$\underset{v\geq 0}{max}\;{\bar{h}}_{k}\left(v,\beta \right)={\left(\frac{k}{2\beta }\right)}^{\frac{k}{2}}e^{-\frac{k}{2}}.$$

of the function ${\bar{h}}_{k}\left(v,\beta \right)=v^{k}e^{-\beta v^{2}}$, grows or decreases rapidly with k, depending on the value of parameter β, the real relaxation spectrum H(v) can be expanded into a series of normalized basis functions:

(8) $${\overset{=}{h}}_{k}\left(v,\beta \right)={\left(\frac{2\beta e}{k}\right)}^{\frac{k}{2}}v^{k}e^{-\beta v^{2}},\;k=1,2,\ldots ,$$

with the first function

$${\overset{=}{h}}_{0}\left(v,\beta \right)=e^{-\beta v^{2}},$$

as follows

(9) $$H\left(v\right)=\sum_{k=0}^{\infty }g_{k}{\overset{=}{h}}_{k}\left(v,\beta \right),$$

where $g_{k}$ are constant model parameters. Since, for the spectra of relaxation times of real materials, the asymptotic property that ℋ(τ)→0 as τ→∞ holds, having in mind the relation $H\left(v\right)=ℋ\left(\frac{1}{v}\right)$, the spectrum of relaxation frequencies tends to zero as the relaxation frequency approaches zero from above, i.e., H(v)→0, as $v\to 0^{+}$. Since ${\overset{=}{h}}_{0}\left(0,\beta \right)=1$, while ${\overset{=}{h}}_{k}\left(0,\beta \right)=0$ for k≥1, the first basis function can be neglected in the series expansion (9). Simultaneously, for practical reasons, it is convenient to replace the infinite summation in (9) with a finite one of K terms, from 1 to K. Therefore, the spectrum H(v) is approximated by a model of the form:

(10) $$H\left(v\right)=\sum_{k=0}^{\infty }g_{k}{\overset{=}{h}}_{k}\left(v,\beta \right),$$

where the new basis functions

(11) $$h_{k}\left(v,\beta \right)={\left(\frac{2\beta e}{k+1}\right)}^{\frac{k+1}{2}}v^{k+1}e^{-\beta v^{2}},\;k=0,1,2,\ldots ,$$

were created as a result of renumbering of ${\overset{=}{h}}_{k}\left(v,\beta \right)$ (8), to unify the presentation of both spectrum models, (5) and (10). According to Equation (2) the respective model of the relaxation modulus G(t) is described by:

(12) $$G_{K}\left(t,\beta \right)=\int_{0}^{\infty }\frac{H_{K}\left(v,\beta \right)}{v}e^{-tv}dv=\sum_{k=0}^{K-1}g_{k}\phi_{k}\left(t,\beta \right),$$

where

(13) $$\phi_{k}\left(t,\beta \right)=\int_{0}^{\infty }\frac{h_{k}\left(v,\beta \right)}{v}e^{-tv}dv.$$

The basis functions $\phi_{k}\left(t,\beta \right)$ (13) of the model (12) are given by compact recursive-analytical formulas specified by the following theorem proved in Appendix A.1.

**Theorem** **2.** *Let* β>0, k≥0 *and* t≥0*. Then the basis functions* $\phi_{k}\left(t,\beta \right)$ *(13) are described by the recursive formula*

(14) $$\phi_{k+1}\left(t,\beta \right)=e\;{\left(\frac{k}{k+2}\right)}^{\frac{k+2}{2}}\left[\phi_{k-1}\left(t,\beta \right)-\frac{1}{\sqrt{2\beta e}\;\sqrt{k}}{\left(\frac{k+1}{k}\right)}^{\frac{k+1}{2}}t\phi_{k}\left(t,\beta \right)\right],$$

*for* k≥1*, starting with*

(15) $$\phi_{0}\left(t,\beta \right)=\sqrt{\frac{\pi e}{2}}{\;e}^{\frac{t^{2}}{4\beta }}\;erfc\left(\frac{t}{2\sqrt{\beta }}\right),$$

*and*

(16) $$\phi_{1}\left(t,\beta \right)=\frac{e}{2}\;\left[1-\frac{1}{\sqrt{2\beta e}}t\;\phi_{0}\left(t,\beta \right)\right],$$

*where the complementary error function* erfc(x) *is defined by [39]:*

(17) $$erfc\left(x\right)=\frac{2}{\sqrt{\pi \;}\;}\int_{x}^{\infty }e^{-z^{2}}dz.$$

Thus, the problem of approximating of the continuous spectrum H(v) by finite series $H_{K}\left(v,\beta \right)$ (10) is reduced to problem of the relaxation modulus G(t) approximation by finite linear combination $G_{K}\left(t,\beta \right)$ (12) of the functions $\phi_{k}\left(t,\beta \right)$ (14)–(16) based on complementary error function $erfc\left(\frac{t}{2\sqrt{\beta }}\right)$. The basis functions $h_{k}\left(v,\beta \right)$ and $\phi_{k}\left(t,\beta \right)$ are dimensionless. A few first basis functions $h_{k}\left(v,\beta \right)$ (11) are shown in Figure 1 for two different values of β; the corresponding functions $\phi_{k}\left(t,\beta \right)$ (14)–(16) are plotted in Figure 2. It is seen from Figure 1 that the maximum of each scaled basis function $h_{k}\left(v,\beta \right)$ is equal one; however, the relaxation frequency $v^{max}$ yielding to the maximum, for a fixed β, depends on the index k according to the formula $v^{max}=\sqrt{\frac{k+1}{2\beta }}$, i.e., grows with k. This means that increasing the number of model components K will allow for good modeling of multimodal spectra. However, modeling of such spectra requires a large number of model components, which is confirmed by Example 2 presented below. Reducing the time-scale factor β shifts the spectrum maxima towards larger relaxation frequencies. In turn, from Figure 2, it is seen that the Debye decay monotonicity of basis functions for the relaxation modulus model is in good agreement with the courses of the relaxation modulus obtained in an experiment for real polymers; for example, elastic polyacrylamide hydrogels [28] (Figures 2a,b, 4a, A5, A7, and A8a).

#### 2.3.1. Positive Definiteness of the Basis Functions

The basis functions of the relaxation frequency spectrum and modulus models are positive definite. Since, for $h_{k}\left(v,\beta \right)$ (11) this property is obvious, the positive definiteness of the functions $\phi_{k}\left(t,\beta \right)$ (14)–(16) directly results from their definition (13). 

#### 2.3.2. Monotonicity of the Basis Functions

The functions $h_{k}\left(v,\beta \right)$ (11) have a global maximum equal to 1. In view of positive definiteness of basis functions $\phi_{k}\left(t,\beta \right)$ (14)–(16), conclusion on their monotonicity results directly from differential property (A1), derived in the Appendix A.1. They are monotonically decreasing for any t≥0. 

#### 2.3.3. Asymptotic Properties of the Basis Functions

Function $\phi_{0}\left(t,\beta \right)$ (15), and whence, in view of (16) and (14), for any k≥1 the basis functions $\phi_{k}\left(t,\beta \right)$, depend on the exponential multiplier $e^{t^{2}/4\beta }$, which rapidly moves towards infinity with growing time t. Therefore, the asymptotic properties of the basis functions (14)–(16) must be analyzed. In Appendix A.2, the following result is derived.

**Theorem** **3.** *Let* β>0, k≥0 *and* t≥0*. The basis functions* $\phi_{k}\left(t,\beta \right)$ *(13) described by the formulas (14)–(16) are such that*

(18) $$\underset{t\to \infty \;}{lim\;}\phi_{k}\left(t,\beta \right)=0,\;\;k=0,1,2,\ldots ,$$

(19) $$\underset{t\to \infty \;}{lim\;}t\phi_{k}\left(t,\beta \right)=0,\;k=1,2,\ldots ,$$

*while*

(20) $$\underset{t\to \infty \;}{lim\;}t\phi_{0}\left(t,\beta \right)=\sqrt{2e\beta \;},$$

*and*

(21) $$\underset{t\to \infty \;}{lim\;}t^{2}\phi_{k}\left(t,\beta \right)=0,\;k=2,3,\ldots .$$

Despite these properties, in numerical computations, the limited values of $\phi_{k}\left(t,\beta \right)$ can be guaranteed only for $t\leq t_{upp}$, where $t_{upp}$ depends on the maximal real number accessible in the computing environment. For example, in Matlab the largest finite floating-point number in IEEE double precision $realmax≅\;1.7977\cdot 10^{308}$. Whence, in view of Equation (15), the range of numerical applicability of the model in the time domain is such that $e^{t^{2}/4\beta }\leq realmax$, i.e.,

(22) $$t\leq t_{upp}=2\sqrt{\beta \ln\left(realmax\right)}≅53.2834\sqrt{\beta }.$$

#### 2.3.4. Ranges of Applicability

In models (10) and (12), the parameter β>0 is a square of the time-scaling factor. The following rule applies: the larger the parameter β, the greater the relaxation times, the lower the relaxation frequencies. The above is illustrated by Figure 1 and Figure 2. Following [12,23], upon the basis functions $\phi_{k}\left(t,\beta \right)$ course, the range of applicability is specified as the time t, for which the first K functions $\phi_{k}\left(t,\beta \right)$ no longer permanently exceeds, i.e., for any θ>t, ε=0.5% of its maximum value. Specifically,

(23) $$t_{app}\left(\beta \right)=\underset{0\leq k\leq K-1}{max}\underset{t>0}{min}\;\left\{t:\left|\phi_{k}\left(\theta ,\beta \right)\right|\leq 0.005\cdot \phi_{kmax}\left(\beta \right)\;\mathrm{for}\;\mathrm{any}\;\theta \geq \;t\right\},$$

where

$$\phi_{kmax}\left(\beta \right)=\underset{t\geq 0}{max}\;\left|\phi_{k}\left(t,\beta \right)\right|.$$

Similarly, in [23], the range of applicability specified directly for the relaxation frequencies v was defined based on the variability of the basis functions $h_{k}\left(v,\beta \right)$, i.e.,

(24) $$v_{app}\left(\beta \right)=\underset{0\leq k\leq K-1}{max}\underset{v>0}{min}\;\left\{\tau :\left|h_{k}\left(\vartheta ,\beta \right)\right|\leq 0.005\cdot h_{kmax}\left(\beta \right)\;\mathrm{for}\;\mathrm{any}\;\vartheta \geq \;v\right\},$$

with $h_{kmax}\left(\beta \right)$ defined by

$$h_{kmax}\left(\beta \right)=\underset{v\geq 0}{max}\;\left|h_{k}\left(v,\beta \right)\right|.$$

The values of $t_{app}\left(\beta \right)$ (23) and $v_{app}\left(\beta \right)$ (24) for different factors β are summarized in Table 1 for K=5 and K=12. For K=6÷11 the same data are given in Table A1 in Appendix B. 

A review of the data shows that the larger the parameter β, the larger the time range $t_{app}\left(\beta \right)$ is and the smaller the frequency range $v_{app}\left(\beta \right)$. The number of model summands K affects the frequency range, slightly increasing $v_{app}\left(\beta \right)$ with increasing K, for fixed β. At the same time, K does not affect the relaxation time range, because it is the first basis function $\phi_{0}\left(t,\beta \right)$ that determines $t_{app}\left(\beta \right)$, see Figure 2. 

### 2.4. Identification Task

Identification consists in selecting, within the chosen class of models given by Equations (5) and (6) or Equations (10) and (12), of such a model, which ensures the best approximation to the measurement data. To unify the description, we will denote the models $G_{K}\left(t,\alpha \right)$ (6) and $G_{K}\left(t,\beta \right)$ (12) of the relaxation modulus, together, as $G_{M}\left(t\right)$. 

For linear viscoelastic materials, the relaxation modulus is the stress, which is induced in the material when the unit step strain is imposed [3,40]. However, it is impossible to apply a step strain in experiments; loading is never performed infinitely fast [41,42,43]. Therefore, the relaxation modulus is not directly accessible by means of a straightforward measurement method and is usually recovered from the experimental data of the stress relaxation process history collected in non-ideal stress relaxation tests. In such two-phase stress relaxation tests, the strain increases over the loading time interval until a predetermined strain is reached, after which the strain is held constant. Different methods have been proposed during the last few decades for the relaxation modulus determination using the stress data histories from non-ideal relaxation tests [42,44,45,46,47,48]. The backward recursive method developed by Lee and Knauss [42], the differential rule proposed by Sorvari and Malinen [44], both addressed to the case of constant loading rate, and the general method proposed by Zapas and Phillips [45], where the ‘true’ relaxation time is delayed of half loading time, are most often cited. For detailed references and an overview, see [41,43,47].

Therefore, suppose a certain identification experiment (stress relaxation test [3,28,40]) resulted in a set of measurements of the relaxation modulus $\left\{\bar{G}\left(t_{i}\right)=G\left(t_{i}\right)+z\left(t_{i}\right)\right\}$ at the sampling instants $t_{i}\geq 0$, i=1,…,N, where $z\left(t_{i}\right)$ is the measurement noise. It is assumed that the number of measurements N≥K. As a measure of the model’s accuracy the quadratic index is taken

(25) $$Q_{N}\left(g_{K}\right)=\sum_{i=1}^{N}{\left[\bar{G}\left(t_{i}\right)-G_{M}\left(t_{i}\right)\right]}^{2}=\|{{\bar{G}}_{N}-\Phi_{N,K}g_{K}\|}_{2}^{2},$$

where $g_{K}={\left[\begin{array}{ccc} g_{0} & \cdots & g_{K-1} \end{array}\right]}^{T}$ is an K-element vector of unknown coefficients of the models (5) and (6) or (10) and (12); ${\left\|\cdot \right\|}_{2}$ denotes the square norm in the real Euclidean space ${ℛ}^{N}$. The matrix $\Phi_{N,K}$ is composed of the basis functions $\varphi_{k}\left(t,\alpha \right)$ (7) or $\phi_{k}\left(t,\beta \right)$ (14)–(16) as follows

(26) $$\Phi_{N,K}=\left[\begin{array}{ccc} \varphi_{0}\left(t_{1},\alpha \right) & \cdots & \varphi_{K-1}\left(t_{1},\alpha \right)\\ \vdots & \ddots & \vdots \\ \varphi_{0}\left(t_{N},\alpha \right) & \cdots & \varphi_{K-1}\left(t_{N},\alpha \right) \end{array}\right]\;\mathrm{or}\;\Phi_{N,K}=\left[\begin{array}{ccc} \phi_{0}\left(t_{1},\beta \right) & \cdots & \phi_{K-1}\left(t_{1},\beta \right)\\ \vdots & \ddots & \vdots \\ \phi_{0}\left(t_{N},\beta \right) & \cdots & \phi_{K-1}\left(t_{N},\beta \right) \end{array}\right]$$

and ${\bar{G}}_{N}$ is the vector of relaxation modulus measurements, i.e., ${\bar{G}}_{N}={\left[\begin{array}{ccc} \bar{G}\left(t_{1}\right) & \cdots & \bar{G}\left(t_{N}\right) \end{array}\right]}^{T}$. 

For real physical materials, the relaxation spectra ℋ(τ) and H(v) are non-negative for any τ≥0 and v≥0. Thus, the requirement that the respective models ${ℋ}_{K}\left(\tau ,\alpha \right)$ (5) and $H_{K}\left(v,\beta \right)$ (10) are also non-negative is natural. The basis functions of both classes of models are non-negative. Therefore, if the model parameters are such that $g_{k}\geq 0$ for any k=0,…,K−1, then the models ${ℋ}_{K}\left(\tau ,\alpha \right)$ and $H_{K}\left(v,\beta \right)$ are non-negative too. The restriction that the model parameters are non-negative is sufficient, but not necessary condition for the non-negativity of the spectrum models. Thus, the optimal identification of non-negative relaxation spectrum models defined by Equations (5) and (6) or Equations (10) and (12) consists in determining the non-negative model parameters minimizing the index $Q_{N}\left(g_{K},\alpha \right)$, i.e., in solving the linear least-squares problem with inequality constraints:

(27) $$\underset{g_{K}\geq 0_{K}}{min}\;\;\;\;\;\|{{\bar{G}}_{N}-\Phi_{N,K}\;g_{K}\|}_{2}^{2},$$

where $0_{K}$ is K dimensional zero vector. 

The existence and properties of the solution of the above task depend on the properties of the matrix $\Phi_{N,K}$. Unfortunately, $\Phi_{N,K}$ is usually rank-deficient. The linear-quadratic task (27) is ill-conditioned [34] and when the data are noisy, even small changes of the data ${\bar{G}}_{N}$ would lead to arbitrarily large artefacts in the optimal model parameters. Therefore, the numerical solution of the finite-dimensional problem (27) is fraught with the same difficulties that the original continuous ill-posed problem of the numerical solution of the Fredholm Equations (1) and (2). The fluctuations of the solution of optimization task (27) may be reduced by introducing an additional direct smoothing constraint ${{\|g}_{K}\|}_{2}\leq \kappa$, where a constant $0<\kappa <\|{\bar{g}}_{K}^{N}{\|}_{2}$ estimates the level of smoothness assumed for the model parameters $g_{K}$; ${\bar{g}}_{K}^{N}$ is the normal (minimum Euclidean norm) solution of the original least-squares problem without constraints. As a result, the modified problem of optimal relaxation spectrum identification is obtained: solve minimization task (27) under constraint

(28) $$\|{g_{K}\|}_{2}^{2}\leq \kappa^{2}.$$

Dual approach is applied below to solve the optimization task (27), (28).

## 3. Results and Discussion

In this section, the optimal identification problem (27) with additional smoothing constraint (28), is solved by applying the dual approach. The existence of the solution of dual maximization task is proved. Next, the idea of parametric optimization [49] is applied to solve the dual task. The necessary and sufficient optimality condition for partial dual tasks is derived in the form of the algebraic polynomial equation. Hierarchical two-stage identification scheme, with the solution of the dual maximization task in two levels, is proposed. Their numerical realization and application of the singular value decomposition technique are discussed. A complete computational algorithm is outlined. The analysis of the smoothness of the relaxation spectra models is presented. 

### 3.1. Dual Problem

By introducing Lagrangian multipliers, a vector $\lambda \geq 0_{K}$ and a scalar γ≥0, we can define the Lagrangian for the optimization task (27), (28)

(29) $$L\left(g_{K},\lambda ,\gamma \right)=\|{{\bar{G}}_{N}-\Phi_{N,K}\;g_{K}\|}_{2}^{2}-\lambda^{T}g_{K}+\gamma \left(\|{g_{K}\|}_{2}^{2}-\kappa^{2}\right),$$

where superscript ‘T’ indicates transpose. The multiplier λ aims at providing a fulfillment of the inequality $g_{K}\geq 0_{K}$. The multiplier γ is the price imposed to satisfy the smoothness constraint (28). The Lagranian is a differentiable function of all arguments. 

The dual problem takes the form:

(30) $$\underset{\lambda \geq 0_{K},\;\;\;\gamma \geq 0\;}{max}\;\;L_{D}\left(\lambda ,\gamma \right)=L_{D}\left(\hat{\lambda },\hat{\gamma }\right),$$

where the dual function is defined as follows:

(31) $$L_{D}\left(\lambda ,\gamma \right)=\underset{g_{K}\in {ℛ}^{K}}{min}\;\;L\left(g_{K},\lambda ,\gamma \right)=L\left({\hat{g}}_{K}\left(\lambda ,\gamma \right),\lambda ,\gamma \right).$$

For an arbitrary κ, λ and γ>0, the Lagrangian $L\left(g_{K},\lambda ,\gamma \right)$ is a strictly convex function of $g_{K}$, which has a unique minimum with respect to $g_{K}$ given by:

(32) $${\hat{g}}_{K}\left(\lambda ,\gamma \right)=\Omega_{K,K}\left(\gamma \right)\left(\Phi_{N,K}^{T}{\bar{G}}_{N}+\frac{1}{2}\lambda \right),$$

with symmetric matrix

(33) $$\Omega_{K,K}\left(\gamma \right)={\left(\Phi_{N,K}^{T}\Phi_{N,K}+\gamma I_{K,K}\right)}^{-1},$$

where $I_{K,K}$ is K dimensional identity matrix. Therefore, the dual function defined in Equation (31), by Formulas (29), and (32), after simple algebraic manipulations, can be expressed in compact form as:

(34) $$L_{D}\left(\lambda ,\gamma \right)={\bar{G}}_{N}^{T}{\bar{G}}_{N}-\gamma \kappa^{2}-{\left(\Phi_{N,K}^{T}{\bar{G}}_{N}+\frac{1}{2}\lambda \right)}^{T}\Omega_{K,K}\left(\gamma \right)\left(\Phi_{N,K}^{T}{\bar{G}}_{N}+\frac{1}{2}\lambda \right).$$

Before we solve dual problem (30), we will give the algebraic background of the method. The algebraic formula describing $L_{D}\left(\lambda ,\gamma \right)$ will be used to derive the basic result regarding the existence of a solution to the dual problem.

### 3.2. Algebraic Tools

Following [12,23], the singular value decomposition (SVD) technique [50] is applied here. Let SVD of the N×K dimensional matrix $\Phi_{N,K}$ (26) takes the form [50]:

(35) $$\Phi_{N,K}=U\Sigma \;V^{T},$$

where $\Sigma =diag\left(\sigma_{1},\ldots ,\sigma_{r},0,\ldots ,0\right)\epsilon {ℛ}^{N,K}$ is a diagonal matrix containing the non-zero singular values $\sigma_{1},\ldots ,\sigma_{r}$ of the matrix $\Phi_{N,K}$, matrices $V\in {ℛ}^{K,K}$ and $U\in {ℛ}^{N,N}$ are orthogonal, and $r=rank\left[\Phi_{N,K}\right]<N$. Due to the diagonal structure of Σ and orthogonality of V and U, matrix $\Omega_{K,K}\left(\gamma \right)$ (33) can be expressed as:

(36) $$\Omega_{K,K}\left(\gamma \right)=V\Lambda \left(\gamma \right)\;V^{T},$$

where K×K diagonal matrix Λ(γ) is as follows:

(37) $$\Lambda \left(\gamma \right)={\left(\Sigma^{T}\Sigma +\gamma I_{K,K}\right)}^{-1}=diag\left(\frac{1}{\sigma_{1}^{2}+\gamma },\ldots ,\frac{1}{\sigma_{r}^{2}+\gamma },\frac{1}{\gamma },\ldots ,\frac{1}{\gamma }\right).$$

Whence, the parameter ${\hat{g}}_{K}\left(\lambda ,\gamma \right)$ (32) is given by:

(38) $${\hat{g}}_{K}\left(\lambda ,\gamma \right)=V\Lambda \left(\gamma \right)\left(\Sigma^{T}\;U^{T}{\bar{G}}_{N}+\frac{1}{2}\;V^{T}\lambda \right).$$

### 3.3. Existence of the Dual Problem Solution 

The following proposition, proved in Appendix A.3, is fundamental for the existence of the solution to the optimization task (30).

**Proposition 1.**  *The dual function* $L_{D}\left(\lambda ,\gamma \right)$ *(34), defined by Equation (31), is strictly concave function of both arguments* (λ,γ), *whenever* γ>0.

Now, the existence of the dual problem solution is resolved by the next result proved in Appendix A.4.

**Theorem** **4.** *If the smoothing parameter* κ *is such that*

(39) $$Y_{K}^{T}Y_{K}>\sigma_{1}^{4}\kappa^{2},$$

*where*

(40) $$Y_{K}=\Phi_{N,K}^{T}{\bar{G}}_{N},$$

*then there exists a solution* $\left(\hat{\lambda },\hat{\gamma }\right)$ *of the dual problem (30), such that* $\hat{\gamma }>0$.

### 3.4. Solution of the Dual Problem

Application of the parametric approach [49] to solve the dual problem (30) results in the scheme:

(41) $$\underset{\lambda \geq 0_{K},\;\;\;\gamma \geq 0\;}{max}\;\;L_{D}\left(\lambda ,\gamma \right)=\underset{\lambda \geq 0_{K}}{max}\left[\underset{\gamma \geq 0}{max}\;\;L_{D}\left(\lambda ,\gamma \right)\right]=\underset{\lambda \geq 0_{K}}{max}\left[{\bar{L}}_{D}\left(\lambda \right)\right]={\bar{L}}_{D}\left(\hat{\lambda }\right)$$

where the function ${\bar{L}}_{D}\left(\lambda ,\gamma \right)$ is defined by the following task:

(42) $$\underset{\gamma \geq 0}{max}\;\;L_{D}\left(\lambda ,\gamma \right)=L_{D}\left(\lambda ,\bar{\gamma }\left(\lambda \right)\right)={\bar{L}}_{D}\left(\lambda \right).$$

From the proof of Theorem 4, it follows that, if the condition (39) is satisfied, then for any fixed $\lambda \geq 0_{K}$ the maximum, with respect to γ, of the strictly concave dual function is positive. Therefore, $\frac{\partial }{\partial \gamma }L_{D}\left(\lambda ,\gamma \right)=0$ is the unique necessary and sufficient condition for $\bar{\gamma }\left(\lambda \right)$ optimality in task (42), which, in view of Equations (A19) and (33), immediately yields the following optimality condition for partial dual problem (42).

**Theorem** **5.** *Assume the condition (39) holds. The optimal Lagrange multiplier* $\bar{\gamma }\left(\lambda \right)$ *solves uniquely the optimization task (42) if and only if the following equation is satisfied*

(43) $$\kappa^{2}={\left(\Phi_{N,K}^{T}{\bar{G}}_{N}+\frac{1}{2}\lambda \right)}^{T}{\left(\Phi_{N,K}^{T}\Phi_{N,K}+\bar{\gamma }\left(\lambda \right)I_{K,K}\right)}^{-2}\left(\Phi_{N,K}^{T}{\bar{G}}_{N}+\frac{1}{2}\lambda \right).$$

The above optimality condition means that for any given $\lambda \geq 0_{K}$ and respective optimal $\bar{\gamma }\left(\lambda \right)$, the smoothing constraint (28) is satisfied as an equation for the resulting model parameter ${\hat{g}}_{K}\left(\lambda ,\bar{\gamma }\left(\lambda \right)\right)$ described by (32). For any fixed λ, Equation (43) can be solved by an arbitrary method of solving nonlinear algebraic equations. By Equations (33), (35) and (36), Equation (43) can be expressed as

(44) $$\kappa^{2}={\left(\Sigma^{T}U^{T}\;{\bar{G}}_{N}+\frac{1}{2}\;V^{T}\lambda \right)}^{T}\Lambda^{2}\left(\gamma \right)\left(\Sigma^{T}U^{T}\;{\bar{G}}_{N}+\frac{1}{2}\;V^{T}\lambda \right),$$

where, in view of Equation (37), diagonal K×K matrix:

$$\Lambda^{2}\left(\gamma \right)=diag\left(\frac{1}{{\left(\sigma_{1}^{2}+\gamma \right)}^{2}},\ldots ,\frac{1}{{\left(\sigma_{r}^{2}+\gamma \right)}^{2}},\frac{1}{\gamma^{2}},\ldots ,\frac{1}{\gamma^{2}}\right)$$

Introducing K element vector $w=\Sigma^{T}U^{T}\;{\bar{G}}_{N}+\frac{1}{2}\;V^{T}\lambda$ composed of the elements $w_{k}$, Equation (44) can be rewritten as:

$$\kappa^{2}=\sum_{k=1}^{r}w_{k}^{2}\frac{1}{{\left(\sigma_{k}^{2}+\gamma \right)}^{2}}+\sum_{k=r+1}^{K}w_{k}^{2}\frac{1}{\gamma^{2}}$$

whence equivalent polynomial equation of unknown variable γ follows:

(45) $$0=\sum_{k=1}^{r}w_{k}^{2}\gamma^{2}\prod_{\begin{array}{c} m=1\\ m\neq k \end{array}}^{r}{\left(\sigma_{m}^{2}+\gamma \right)}^{2}+\prod_{m=1}^{r}{\left(\sigma_{m}^{2}+\gamma \right)}^{2}\left[\sum_{k=r+1}^{K}w_{k}^{2}-\kappa^{2}\gamma^{2}\right].$$

To solve Equation (45) of order 2(r+1), in general, any numerical method of solving polynomial equations can be applied [51].

In view of Equations (42) and (34) we have:

(46) $${\bar{L}}_{D}\left(\lambda \right)={\bar{G}}_{N}^{T}{\bar{G}}_{N}-\kappa^{2}\bar{\gamma }\left(\lambda \right)-{\left(\Phi_{N,K}^{T}{\bar{G}}_{N}+\frac{1}{2}\lambda \right)}^{T}\Omega_{K,K}\left(\bar{\gamma }\left(\lambda \right)\right)\left(\Phi_{N,K}^{T}{\bar{G}}_{N}+\frac{1}{2}\lambda \right).$$

In the Appendix A.5, the following formula is derived:

(47) $$\frac{\partial }{\partial \lambda }{\bar{L}}_{D}\left(\lambda \right)=-\Omega_{K,K}\left(\bar{\gamma }\left(\lambda \right)\right)\left(\Phi_{N,K}^{T}{\bar{G}}_{N}+\frac{1}{2}\lambda \right),$$

where $\bar{\gamma }\left(\lambda \right)$ satisfies Equation (43). 

By SVD (35) and Equation (36), function ${\bar{L}}_{D}\left(\lambda \right)$ (46) takes the form:

(48) $${\bar{L}}_{D}\left(\lambda \right)={\bar{G}}_{N}^{T}{\bar{G}}_{N}-\kappa^{2}\bar{\gamma }\left(\lambda \right)-{\left(\Sigma^{T}\;U^{T}{\bar{G}}_{N}+\frac{1}{2}\;V^{T}\lambda \right)}^{T}\Lambda \left(\bar{\gamma }\left(\lambda \right)\right)\left(\Sigma^{T}\;U^{T}{\bar{G}}_{N}+\frac{1}{2}\;V^{T}\lambda \right),$$

while the gradient (47), in view of Equation (36), is equivalently given by:

$$\frac{\partial }{\partial \lambda }{\bar{L}}_{D}\left(\lambda \right)=-V\Lambda \left(\bar{\gamma }\left(\lambda \right)\right)\left(\Sigma^{T}\;U^{T}{\bar{G}}_{N}+\frac{1}{2}\;V^{T}\lambda \right).$$

For solving the optimization task:

(49) $$\underset{\lambda \geq 0_{K}}{max\;}\left[{\bar{L}}_{D}\left(\lambda \right)\right]={\bar{L}}_{D}\left(\hat{\lambda }\right),$$

numerical methods of constrained nonlinear programming [52] can be applied.

### 3.5. Solution of the Smoothed Identification Problem

If the saddle point of the Lagrangian $L\left(g_{K},\lambda ,\gamma \right)$ (29) exists, then the dual approach can be successfully applied to solve the optimization task (27) with the smoothing constraint (28), i.e., to solve the stated identification problem. In the case considered, the existence of a saddle point to the Lagrangian follows immediately from Theorem 1, cases (ii) and (iii) in [53] due to the uniqueness of the minimum of $L\left(g_{K},\lambda ,\gamma \right)$ with respect to $g_{K}$, given by Equation (32). Thus, the vector:

(50) $${\hat{g}}_{K}={\hat{g}}_{K}\left(\hat{\lambda },\hat{\gamma }\right)=\Omega_{K,K}\left(\hat{\gamma }\right)\left(\Phi_{N,K}^{T}{\bar{G}}_{N}+\frac{1}{2}\hat{\lambda }\right),$$

or, equivalently,

(51) $${\hat{g}}_{K}={\hat{g}}_{K}\left(\hat{\lambda },\hat{\gamma }\right)=V\Lambda \left(\hat{\gamma }\right)\left(\Sigma^{T}\;U^{T}{\bar{G}}_{N}+\frac{1}{2}\;V^{T}\hat{\lambda }\right),$$

where $\hat{\gamma }=\bar{\gamma }\left(\hat{\lambda }\right)$, is optimal solution of the optimization task (27), (28), i.e., the vector of the best model parameters. According to Theorem 4, the price $\hat{\gamma }>0$ and, by Equation (43), for optimal ${\hat{g}}_{K}$ (50) we have

(52) $$\|{{\hat{g}}_{K}\|}_{2}^{2}=\kappa^{2},$$

i.e., the smoothness constraint (28) is equally satisfied. 

### 3.6. Two-Level Solution od the Dual Problem

To solve the dual problem (30) according to the optimization task (41), i.e., by successive maximization with respect to γ and λ, the following two-level algorithm can be applied. 

*Lower level of the dual problem solution:*

Given the Lagrange multiplier $\lambda \geq 0_{K}$, find the multiplier $\bar{\gamma }\left(\lambda \right)\geq 0$ maximizing $L_{D}\left(\lambda ,\gamma \right)$ in the optimization task (42) by solving Equation (43). 

*Upper level of the dual problem solution:*

Find the multiplier $\hat{\lambda }\geq 0_{K}$ solving the optimization task (49). 

The resulting pair $\left(\hat{\lambda },\hat{\gamma }\right)$, $\hat{\gamma }=\bar{\gamma }\left(\hat{\lambda }\right)$, solves the dual problem (30). The numerical computations must be arranged hierarchically, i.e., in each iteration of the maximization procedure at the upper level, the algebraic equation (43) must be solved in the lower-level task. The complete computational procedure for determining the dual problem solution and, next, the optimal model of the relaxation spectrum is given below.

### 3.7. Identification Scheme

The determination of the model of the relaxation spectrum involves the following steps.

1. Perform the experiment (stress relaxation test [3,28,40]) and record the measurements $\bar{G}\left(t_{i}\right)$, i=1,…,N, of the relaxation modulus at times $t_{i}\geq 0$.
2. Choose the number K of model components and, depending on the relaxation spectrum recovery problem considered, the time-scaling factor α for identification of relaxation time spectrum or β for spectrum of relaxation frequencies determination, comparing, for different values of α or β, a few first functions from the sequence $\left\{\varphi_{k}\left(t,\alpha \right)\right\}$ or $\left\{\phi_{k}\left(t,\beta \right)\right\}$ with the experiment results $\left\{\bar{G}\left(t_{i}\right)\right\}$.
3. Compute the matrix $\Phi_{N,K}$ (26) and, next, determine SVD (35).
4. Compute $\|{{\bar{g}}_{K}^{N}\|}_{2}$ and choose the constant $0<\kappa <\|{{\bar{g}}_{K}^{N}\|}_{2}$.
5. Determine, in the following two-level computations, the solution $\left(\hat{\lambda },\hat{\gamma }\right)$ of the dual problem (30).
  5.1 Choose the initial multiplier $\lambda^{0}$ for the numerical procedure applied to solve optimization task (49).
  5.2 Let $\lambda^{m}$ be the m-th iterate in the numerical procedure solving (49). For $\lambda =\lambda^{m}$ solve the Equation (43) according to the chosen numerical procedure and determine $\bar{\gamma }\left(\lambda^{m}\right)$. Polynomial Equation (45) can be solved instead of Equation (43).
  5.3 Using $\bar{\gamma }\left(\lambda^{m}\right)$, compute the new multiplier $\lambda^{m+1}$, being the next approximation of $\hat{\lambda }$, according to the numerical procedure selected to solve the task (49), with the maximized index ${\bar{L}}_{D}\left(\lambda \right)$ given by Equation (46) or, equivalently, by Equation (48). If, for $\lambda^{m+1}$, the stopping rule of the chosen numerical procedure is satisfied, i.e.,
    $$\|{\lambda^{m+1}-\lambda^{m}\|}_{2}\leq \varepsilon_{1}$$
    or
    $$\left|{\bar{L}}_{D}\left(\lambda^{m+1}\right)-{\bar{L}}_{D}\left(\lambda^{m}\right)\right|\leq \varepsilon_{2},$$
    where $\varepsilon_{1}$ and $\varepsilon_{2}$ are preselected small positives, take $\hat{\lambda }=\lambda^{m}$ and $\hat{\gamma }=\bar{\gamma }\left(\lambda^{m}\right)$ as the solution to the dual problem (30), and go to Step 6. Otherwise return to Step 5.2 and continue the computations for $\lambda =\lambda^{m+1}$.
6. Compute the vector of the optimal model parameters ${\hat{g}}_{K}$ according to Equation (50) or (51) and, depending on the spectrum recovery problem, determine the optimal model of the relaxation time spectrum given by:
  (53) $${\hat{ℋ}}_{K}\left(\tau ,\alpha \right)=\sum_{k=0}^{K-1}{\hat{g}}_{k}h_{k}\left(\tau ,\alpha \right),$$
  or optimal model of the spectrum of relaxation frequencies described by:
  (54) $${\hat{H}}_{K}\left(v,\beta \right)=\sum_{k=0}^{K-1}{\hat{g}}_{k}h_{k}\left(v,\beta \right),$$
  where ${\hat{g}}_{k}$ are elements of the vector ${\hat{g}}_{K}$.

**Figure 3:**
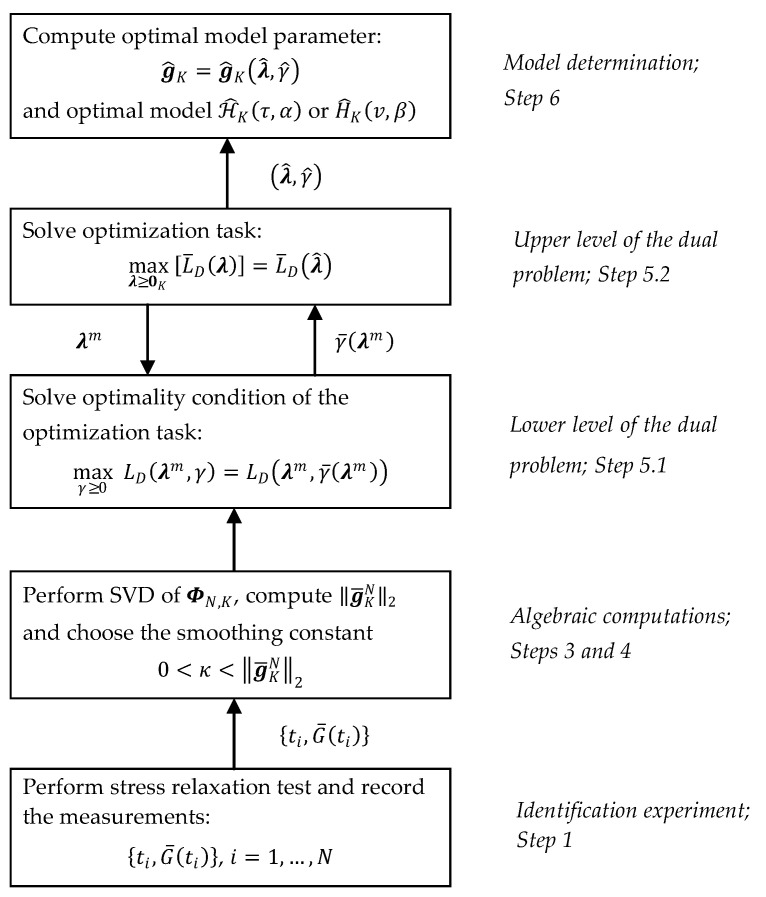
Hierarchical structure of the tasks solved for determination of the optimal spectrum relaxation model.

The schematic framework of the above procedure and communication between the two levels solving dual problem and the remaining tasks and relaxation test experiment are shown in Figure 3.

### 3.8. Remarks

1. The SVD (35) of the matrix $\Phi_{N,K}$, of computational complexity $O\left(NK^{2}\right)$ [50], must be computed only once in Step 3 and should not be repeated during the two-level computations of Step 5.
2. The matrix $\Phi_{N,K}$ depends on the choice of the basis functions as well as the measurement points $t_{i}$; however, it does not depend on the relaxation modulus measurements $\bar{G}\left(t_{i}\right)$. Thus, when the identification scheme is applied for successive samples of the same material, Step 3 should not be repeated while the same time instants $t_{i}$ are kept and the same model parameters α or β and K are used (selected in Step 2).
3. Since the normal solution ${\bar{g}}_{K}^{N}=\Phi_{N,K}^{†}{\bar{G}}_{N}$, where $\Phi_{N,K}^{†}=V\Sigma^{†}U^{T}$ is the Moore–Penrose pseudoinverse [54] of matrix $\Phi_{N,K}$ (35) with K×N matrix $\Sigma^{†}=diag\left(1/\sigma_{1},\ldots ,1/\sigma_{r},0,\ldots ,0\right)$, the norm computed in Step 4 is as follows
  $$\|{{\bar{g}}_{K}^{N}\|}_{2}^{2}=\sum_{i=1}^{r}\frac{\;x_{i}^{2}}{\sigma_{i}^{2}},$$
  where $x_{i}$ are elements of the N dimensional vector $X=U^{T}\;{\bar{G}}_{N}$.
4. The basis functions $\varphi_{k}\left(t,\alpha \right)$ (7) are the products of power of time and the modified Bessel functions of the second kind, while the basis functions $\phi_{k}\left(t,\beta \right)$ (14)–(16) are expressed using complementary error function. The modified Bessel functions of the second kind are accessible, for example, in Matlab as *besselk* function. The function erfc(x) is accessible practically in every computational packets either directly or by the error function erf(x)=1−erfc(x).
5. In the models proposed the parameters α and β are the time-scaling factors. For the relaxation time model the following rule holds, the lower the parameter α is, the greater the relaxation times are [12]. For the relaxation frequency model, the larger the parameter β, the greater the relaxation times, the lower the relaxation frequencies. Through the optimal choice of the scaling factors, the best fit of the model to the experimental data can be achieved. In [12], the hierarchical algorithm with the optimal choice of the time-scaling factor α was presented. However, practically in many cases, the selection of the time-scaling factors in Step 2 based on the data concerning model applicability summarized in Table 1 and Table A1 for factor β and related tables in [12] for factor α or based on the comparison of a few first functions from the sequences $\left\{\varphi_{k}\left(t,\alpha \right)\right\}$ or $\left\{\phi_{k}\left(t,\beta \right)\right\}$ for different values of α or β with the experimentally obtained function $\bar{G}\left(t_{i}\right)$, is quite enough. Similarly, the number K of the models $G_{K}\left(t,\alpha \right)$ (6) or $G_{K}\left(t,\beta \right)$ (12) series elements can be initially evaluated. This rough selection strategy of the model parameters selection was applied in [23]. Thus, the choice of K and α must be carried out *a posteriori*, after the preliminary analysis of the experiment data.
6. Only the values of $\lambda^{m}$ and $\bar{\gamma }\left(\lambda^{m}\right)$, not the related parameter ${\hat{g}}_{K}\left(\lambda^{m},\bar{\gamma }\left(\lambda^{m}\right)\right)$ described by Equation (32), are used in iterations of the numerical procedures solving the dual problem tasks in Step 5. The vector ${\hat{g}}_{K}$ of optimal model parameters is computed only in Step 6.

### 3.9. Smoothness Analysis

The smoothing constraint (28) was introduced to stabilize the resulting vector ${\hat{g}}_{K}$ (50), for which the equality (52) holds. Since the non-negative basis functions $h_{k}\left(\tau ,\alpha \right)$ (3) and $h_{k}\left(v,\beta \right)$ (11) for any arguments are bounded by one, the following inequalities:

$$\underset{\tau \geq 0}{max\;}{\hat{ℋ}}_{K}\left(\tau ,\alpha \right)\leq \sum_{k=0}^{K-1}\left|{\hat{g}}_{k}\right|\;\mathrm{and}\;\underset{v\geq 0}{max\;}{\hat{H}}_{K}\left(v,\beta \right)\leq \sum_{k=0}^{K-1}\left|{\hat{g}}_{k}\right|$$

hold for the optimal models (53) and (54) with arbitrary time-scale factors, which means that the smoothing of the vector of model parameters imply the boundness of the respective relaxation spectra. 

The norms $\|{{ℋ}_{K}\left(\tau ,\alpha \right)\|}_{2}$ and $\|{H_{K}(v,\beta )\|}_{2}$ are also the measures of smoothness of the spectra models, where ${\left\|\cdot \right\|}_{2}$ means here the square norm in $L^{2}(0,\infty )$. Proposition 1 in [Belssel] characterizes $\|{{ℋ}_{K}\left(\tau ,\alpha \right)\|}_{2}$ as the square form of $g_{K}$ with the matrix dependent on α. In the Appendix A.6 the analogous property for the spectrum $H_{K}\left(v,\beta \right)$ is proved.

**Proposition** **2.** *For an arbitrary time-scale factor* β *and arbitrary vector of model parameters* $g_{K}$ *for the relaxation spectrum model* $H_{K}\left(v,\beta \right)$ *(10) we have*

(55) $$\|{H_{K}(v,\beta )\|}_{2}^{2}=\frac{1}{\sqrt{2\beta e}}g_{K}^{T}\Theta g_{K},$$

*where* Θ *is* K×K *symmetric, positive definite, real matrix of the elements:*

(56) $$\theta_{kj}\left(\beta \right)=\frac{{\left(\frac{k+j+3}{k+1}\right)}^{\frac{k+1}{2}}{\left(\frac{k+j+3}{j+1}\right)}^{\frac{j+1}{2}}\sqrt{k+j+3}}{\sqrt{2^{k+j+3}}}\phi_{k+j+2}\left(0,2\beta \right),$$

*where* k,j=0,1,…K−1 *and the values of the basis functions* $\phi_{k}\left(t,\beta \right)$ *(14)–(16) at* t=0 *are as follows*

(57) $$\phi_{2k}\left(0,2\beta \right)={\left(\frac{e}{2k+1}\right)}^{k}\prod_{j=1}^{k-1}\left(2j+1\right)\sqrt{\frac{\pi e}{2\left(2k+1\right)}}$$

*for even indices and *

(58) $$\phi_{2k+1}\left(0,2\beta \right)={\left(\frac{e}{k+1}\right)}^{k+1}\frac{k!}{2}\;$$

*for odd indices, where* k=1,2,…*. Matrix* Θ *is a positive definite*.

The basis functions $\phi_{k}\left(0,2\beta \right)$ (57), (58) for the time t=0 do not depend on the time-scale factor, in fact. Propositions 2 and 3 in [12] specify various useful estimates of $\|{{ℋ}_{K}(\tau ,\alpha )\|}_{2}$, which can be directly applied to obtain the respective estimates of the norm $\|{H_{K}(v,\beta )\|}_{2}$ and, for the optimal models, results in the next property.

**Proposition** **3.** *For an arbitrary time-scale factors* α *and* β *and the vector of optimal model parameters* ${\hat{g}}_{K}$ *(50), for the optimal models* ${\hat{ℋ}}_{K}\left(\tau ,\alpha \right)$ *(53) and* ${\hat{H}}_{K}\left(v,\beta \right)$ *(54) the following upper*:

$$\|{\hat{ℋ}}_{K}{\left(\tau ,\alpha \right)\|}_{2}\leq \frac{1}{\sqrt{2\alpha }}\sqrt{{\bar{\sigma }}_{1}\left(\Gamma_{1}\right)}\|{\hat{g}}_{K}\|{}_{2}=\frac{1}{\sqrt{2\alpha }}\sqrt{{\bar{\sigma }}_{1}\left(\Gamma_{1}\right)}\;\kappa ,$$

(59) $$\|{\hat{H}}_{K}{\left(v,\beta )\right\|}_{2}\leq \frac{1}{\sqrt[{4}]{2\beta e}}\sqrt{{\bar{\sigma }}_{1}\left(\Theta \right)}\|{\hat{g}}_{K}\|{}_{2}=\frac{1}{\sqrt[{4}]{2\beta e}}\sqrt{{\bar{\sigma }}_{1}\left(\Theta \right)}\;\kappa ,$$

*and lower*

$$\|{\hat{ℋ}}_{K}{\left(\tau ,\alpha \right)\|}_{2}\geq \frac{1}{\sqrt{2\alpha }}\sqrt{{\bar{\sigma }}_{min}\left(\Gamma_{1}\right)}\|{\hat{g}}_{K}\|{}_{2}=\frac{1}{\sqrt{2\alpha }}\sqrt{{\bar{\sigma }}_{min}\left(\Gamma_{1}\right)}\;\kappa ,$$

(60) $${\|\hat{H}}_{K}{\left(v,\beta \right)\|}_{2}\geq \frac{1}{\sqrt[{4}]{2\beta e}}\sqrt{{\bar{\sigma }}_{min}\left(\Theta \right)}\|{\hat{g}}_{K}\|{}_{2}=\frac{1}{\sqrt[{4}]{2\beta e}}\sqrt{{\bar{\sigma }}_{min}\left(\Theta \right)}\;\kappa .$$

*bounds hold, where* ${\bar{\sigma }}_{1}\left(\Theta \right)$*,* ${\bar{\sigma }}_{1}\left(\Gamma_{1}\right)$ *are the largest and* ${\bar{\sigma }}_{min}\left(\Theta \right)$*,* ${\bar{\sigma }}_{min}\left(\Gamma_{1}\right)$ *are the minimal singular values of matrix* Θ *(56)–(58) and matrix* $\Gamma_{1}$ *defined in [12] (Equation (53)).*

The square roots of the singular values ${\bar{\sigma }}_{1}\left(\Theta \right)$ and ${\bar{\sigma }}_{min}\left(\Theta \right)$ for K=5,6, …12 are summarized in Table 2. Since $\sqrt{{\bar{\sigma }}_{1}\left(\Theta \right)}$ grows with K, the greater the number of model summands are, the greater time scaling factor should be, to achieve pre-assumed multiplier $\sqrt{{\bar{\sigma }}_{1}\left(\Theta \right)}/\sqrt[{4}]{2\beta e}$ in the estimation (59). However, this increase is relatively much weaker than in the case of the model ${ℋ}_{K}\left(\tau ,\alpha \right)$ (53). Similarly, as in the case of ${ℋ}_{K}\left(\tau ,\alpha \right)$ (for details, see [12]), estimation (60) is useful only for small K and small time-scale factors. Thus, the smoothness of the vector ${\hat{g}}_{K}$ (50) of model parameters guarantees that the fluctuations of the respective spectra of relaxation ${\hat{ℋ}}_{K}\left(\tau ,\alpha \right)$ and ${\hat{H}}_{K}\left(v,\beta \right)$ are also bounded. The time-scale factors α and β affect the smoothness of the models. 

### 3.10. Examples

**Figure 4:**
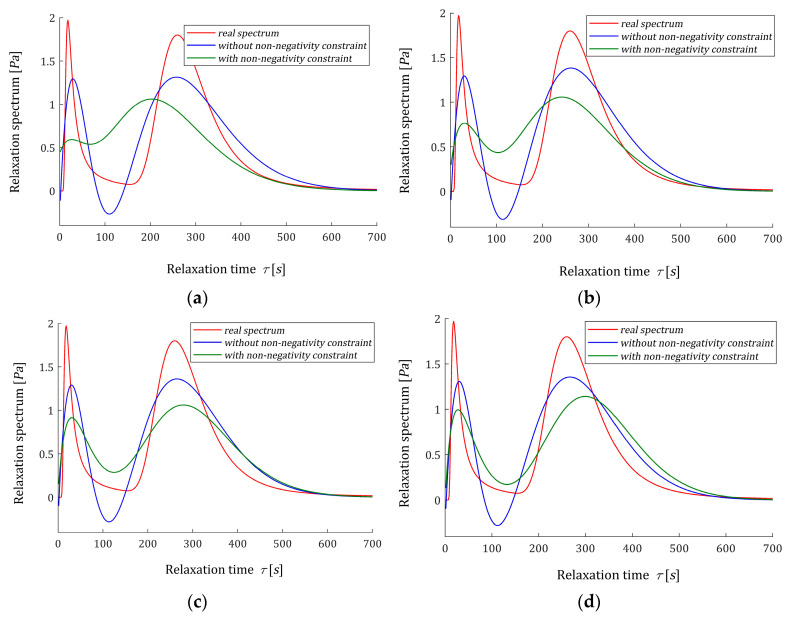
Relaxation time spectrum H(τ) (63) (solid red line) from Example 1 and the corresponding optimal models determined without non-negativity constraint ${\tilde{ℋ}}_{K}\left(\tau ,\alpha_{opt}\right)$ (61) (blue line) and with non-negativity constraint ${\hat{ℋ}}_{K}\left(\tau ,\alpha_{opt}\right)$ (54) (green line) for K summands of the model: (**a**) K=6; (**b**) K=8; (**c**) K=10; (**d**) K=12.

Three examples are presented below. In two examples, the relaxation spectra described by the double-mode Gauss-like distributions are considered since spectra of this type describe the viscoelastic properties of various polymers: [27] (Figures 4b and 8b), polyacrylamide gels [28] (Figure A4), cold gel-like emulsions stabilized with bovine gelatin [29], fresh egg white-hydrocolloids foams [31] (Figures 6 and 14) and are tested when developing new identification methods; for example, in [8] (Figure 2), [9] (Figures 9, 11 and 17) and [10] (Figures 2, 3, 6, 7–11 and 14). In the third example, one-mode Gauss distribution was taken; typical, for example, for relaxation spectra of some native starch gels [26] (Figures 6b, 7 and 9a).

As in [12], in all examples for numerical experiment N=5000, sampling instants $t_{i}$ were generated with the constant period in the time interval T=[0,T] seconds and additive measurement noises $z\left(t_{i}\right)$ were selected independently by random choice with uniform distribution on the interval [−0.005, 0.005] Pa. The real spectra and modulus and the basis functions $h_{k}\left(\tau ,\alpha \right)$ (3), $h_{k}\left(v,\beta \right)$ (11) of the spectra models and $\varphi_{k}\left(t,\alpha \right)$ (7), $\phi_{k}\left(t,\beta \right)$ (14)–(16) of the modulus models were simulated in Matlab R2022a using the special functions *besselk* and *erfc*. For the singular value decomposition procedure, *svd* was applied. 

The relaxation time and frequencies models are determined in the class of models defined by ${ℋ}_{K}\left(\tau ,\alpha \right)$ (5) and $H_{K}\left(v,\beta \right)$ (10). In all examples, the same workflow is applied. First, the optimal models were determined by a two-level regularized least-squares identification scheme proposed in the previous paper [12], i.e., neglecting the non-negativity requirement. This means that, in particular, the optimal time-scale factors $\alpha_{opt}$ and $\beta_{opt}$ and the optimal regularized model parameter vectors ${\tilde{g}}_{K}$ were determined resulting in the unconstrained, i.e., determined without non-negativity constraint, models of relaxation time:

(61) $${\tilde{ℋ}}_{K}\left(\tau ,\alpha_{opt}\right)=\sum_{k=0}^{K-1}{\tilde{g}}_{k}h_{k}\left(\tau ,\alpha_{opt}\right),$$

and relaxation frequencies

(62) $${\tilde{H}}_{K}\left(v,\beta_{opt}\right)=\sum_{k=0}^{K-1}{\tilde{g}}_{k}h_{k}\left(v,\beta_{opt}\right),$$

where ${\tilde{g}}_{k}$ are elements of the vector ${\tilde{g}}_{K}$. 

Next, for the optimal factors $\alpha_{opt}$ and $\beta_{opt}$, the best models with the optimal parameters ${\hat{g}}_{K}\geq 0_{K}$ are determined with the non-negativity requirement using the scheme proposed above. As a result, the relaxation spectra ${\hat{ℋ}}_{K}\left(\tau ,\alpha \right)$ (54) and ${\hat{H}}_{K}\left(v,\beta \right)$ (54) were obtained for time scale factors $\alpha =\alpha_{opt}$ and $\beta =\beta_{opt}$ with the non-negative optimal parameters ${\hat{g}}_{K}$ (50). The smoothing parameter κ was selected several times until a satisfactory accuracy of the fit of the model to the experimental data was obtained. Since some elements of the vectors ${\tilde{g}}_{K}$ are negative, i.e., the respective components of the models (61) and (62) are negative too, κ smaller than the norm $\|{{\tilde{g}}_{K}\|}_{2}$ are applied.

#### 3.10.1. Example 1

Consider viscoelastic material of relaxation spectrum described by the double-mode Gauss-like distribution considered in [12,27]:

(63) $$ℋ\left(\tau \right)=\left[\vartheta_{1}e^{-{\left(\frac{1}{\tau }-m_{1}\right)}^{2}/q_{1}}+\vartheta_{2}e^{-{\left(\frac{1}{\tau }-m_{2}\right)}^{2}/q_{2}}\right]/\tau ,$$

inspired by polyethylene data from [27], especially HDPE 1 sample from [27] (Table 1 and Figure 8b), where the parameters are as follows [12]: $\vartheta_{1}=467\;\mathrm{Pa}\cdot s$, $m_{1}=0.0037\;s^{-1}$, $q_{1}=1.124261\times 10^{-6}\;s^{-2}\;$, $\vartheta_{2}=39\;\mathrm{Pa}\cdot s$, $m_{2}=0.045\;s^{-1}$ and $q_{2}=1.173\times 10^{-3}\;s^{-2}$. It is shown in [12] that the related real relaxation modulus is

(64) $$G\left(t\right)=\frac{\sqrt{\pi }}{2}\left[\vartheta_{1}\sqrt{q_{1}}\;e^{\frac{1}{4}t^{2}q_{1}-m_{1}t}erfc\left(\frac{\frac{1}{2}tq_{1}-m_{1}}{\sqrt{q_{1}}}\right)+\vartheta_{2}\sqrt{q_{2}}\;e^{\frac{1}{4}t^{2}q_{2}-m_{2}t}erfc\left(\frac{\frac{1}{2}tq_{2}-m_{2}}{\sqrt{q_{2}}}\right)\right].$$

**Figure 5:**
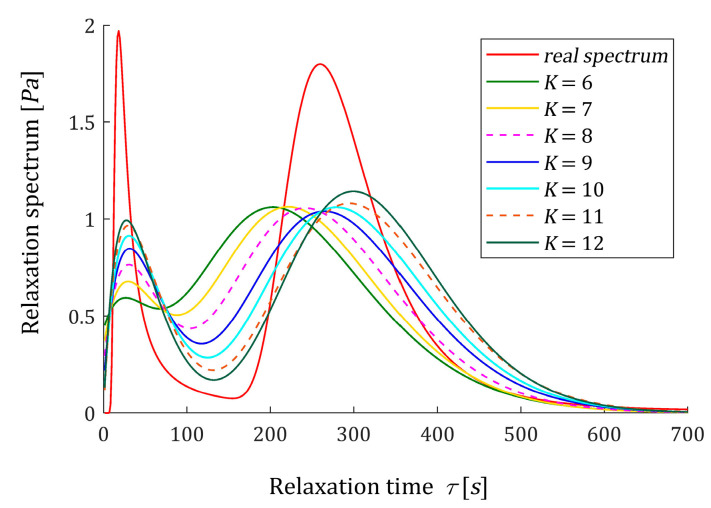
Relaxation time spectrum H(τ) (63) from Example 1 and the optimal non-negative models ${\hat{ℋ}}_{K}\left(\tau ,\alpha_{opt}\right)$ (54) for K=6,…,12 summands of the model.

**Figure 6:**
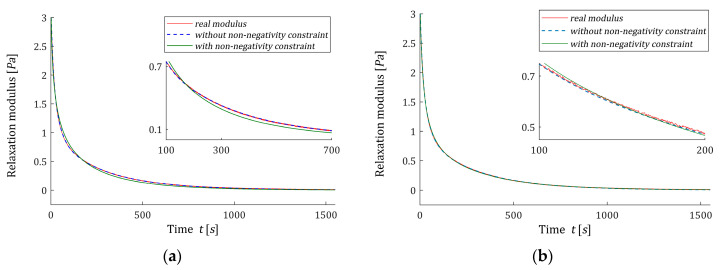
The measurements $\bar{G}\left(t_{i}\right)$ of the real relaxation modulus G(t) (64) (red points) from Example 1 and the optimal approximated models determined without non-negativity constraint ${\tilde{G}}_{K}\left(t,\alpha_{opt}\right)$ and with non-negativity constraint ${\hat{G}}_{K}\left(t,\alpha_{opt}\right)$ for K model summands: (**a**) K=8; (**b**) K=12.

**Figure 7:**
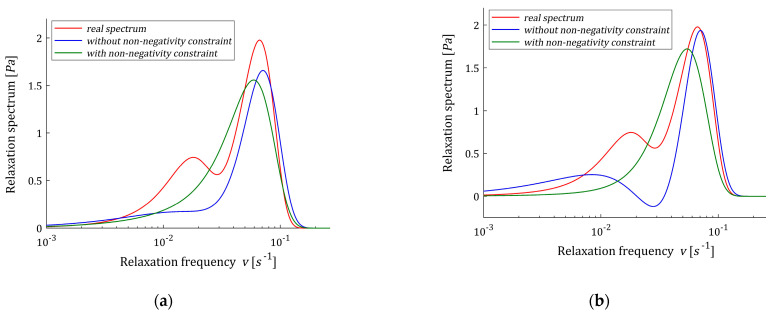

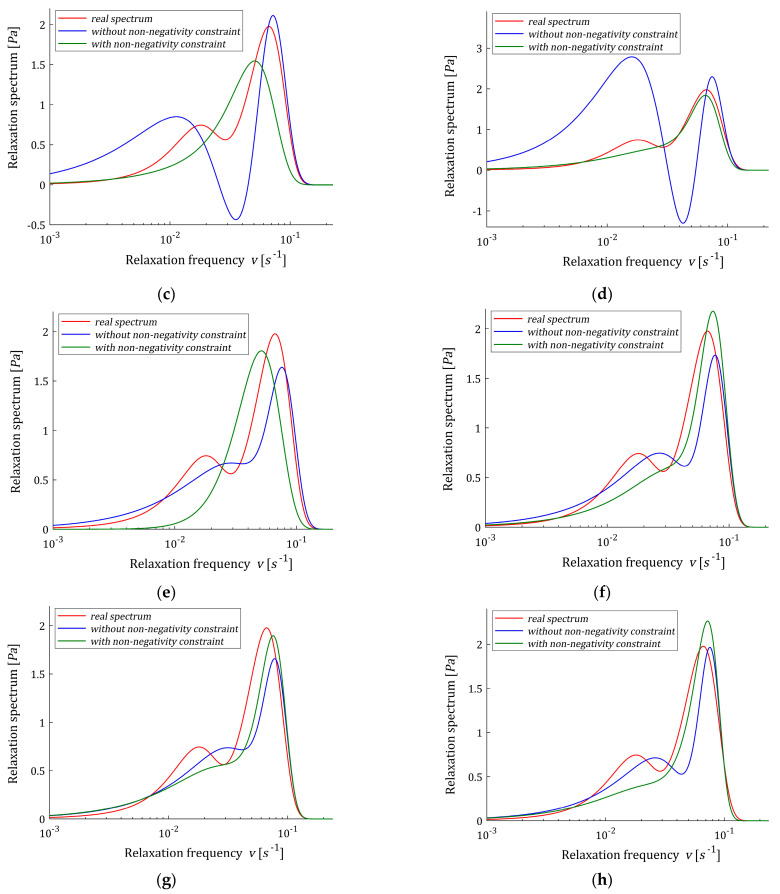
Relaxation frequency spectrum H(v) (65) (solid red line) from Example 2 and the corresponding optimal models determined without non-negativity constraint ${\tilde{H}}_{K}\left(v,\beta_{opt}\right)$ (62) and with non-negativity constraint ${\hat{H}}_{K}\left(v,\beta_{opt}\right)$ (54) (green line) for K summands of the model: (**a**) K=6; (**b**) K=8; (**c**) K=10; (**d**) K=12; (**e**) K=14; (**f**) K=16; (**g**) K=18; (**h**) K=20.

Following [12], the time interval T=[0,1550] seconds is assumed for numerical experiments. In [12], the optimal models ${\tilde{ℋ}}_{K}\left(\tau ,\alpha_{opt}\right)$ (61) with the parameter vectors ${\tilde{g}}_{K}$ and time-scaling factors $\alpha_{opt}$ were determined for K=3,4,…,10. Detailed data, including $\alpha_{opt}$, ${\tilde{g}}_{K}$, regularization parameters $\lambda_{GCV}\left(\alpha_{opt}\right)$, the square norms $\|{{\tilde{g}}_{K}\|}_{2}$ and $\|{{\tilde{ℋ}}_{K}(\tau ,\alpha_{opt})\|}_{2}$, as the measures of the solution smoothness, and mean square identification index $Q_{N}\left({\tilde{g}}_{K}\right)/N$ were summarized in [12] and (Tables 3 and A3). Only $\alpha_{opt}$, $Q_{N}\left({\tilde{g}}_{K}\right)/N$ and $\|{{\tilde{g}}_{K}\|}_{2}$ are rewritten here in Table 3; the last two to compare with respective data for the constrained non-negative model ${\hat{ℋ}}_{K}\left(\tau ,\alpha_{opt}\right)$ (54). The vectors ${\tilde{g}}_{K}$ are given in [12] (Table A3), from which it can be seen that some of their elements are negative. For K=3 one element, for K=4 two elements, for K=5,7,8,9 three elements and for K=6,10,11,12 four elements are negative. Also, the spectra ${\tilde{ℋ}}_{K}\left(\tau ,\alpha_{opt}\right)$ (61) are negative for some ranges of the relaxation frequencies, see [12] (Figures 4 and 7a) and also Figure 4, below. The non-negative optimal vectors ${\hat{g}}_{K}$ are given in Table A2 in Appendix B. Only two or three elements of these vectors are non-zero, the corresponding elements of the Lagrange multipliers vectors $\hat{\lambda }$ are obviously zero. Other numerical data for optimal non-negative models ${\hat{ℋ}}_{K}\left(\tau ,\alpha_{opt}\right)$, i.e., square norm $\|{{\tilde{g}}_{K}\|}_{2}$, identification index $Q_{N}\left({\hat{g}}_{K}\right)/N$ and the Lagrange multiplier $\hat{\gamma }$ are given in the last columns of Table 3. Figure 4 illustrated the course of the real spectrum ℋ(τ) (63), the unconstrained model ${\tilde{ℋ}}_{K}\left(\tau ,\alpha_{opt}\right)$ (61) (blue line) and non-negative model ${\hat{ℋ}}_{K}\left(\tau ,\alpha_{opt}\right)$ (54) (green line) for K=6,8,10,12. The non-negative models ${\hat{ℋ}}_{K}\left(\tau ,\alpha_{opt}\right)$ are summarized in Figure 5 for K=6,7,…,12. In Figure 6, the models of the relaxation modulus ${\tilde{G}}_{K}\left(t,\alpha_{opt}\right)$ and ${\hat{G}}_{K}\left(t,\alpha_{opt}\right)$ corresponding to ${\tilde{ℋ}}_{K}\left(\tau ,\alpha_{opt}\right)$ (61) and ${\hat{ℋ}}_{K}\left(\tau ,\alpha_{opt}\right)$ (54), respectively, computed according to Equation (6) are plotted for K=8,12, where the measurements $\bar{G}\left(t_{i}\right)$ of the real modulus G(t) (64) are also marked. The optimal models ${\tilde{G}}_{K}\left(t,\alpha_{opt}\right)$ have been better fitted to the experimental data than ${\hat{G}}_{K}\left(t,\alpha_{opt}\right)$, thus ${\tilde{G}}_{K}\left(t,\alpha_{opt}\right)$ practically coincide with the measurement points. The deterioration of the identification index for the non-negative models changes from 5.97 times for K=12 to 65.56 times for K=5.

#### 3.10.2. Example 2

Consider again the double-mode Gauss-like distribution described by equation (63). Now the parameters are: $\vartheta_{1}=42.2\;\mathrm{Pa}\cdot s$, $m_{1}=0.013012\;s^{-1}$, $q_{1}=1.25\times 10^{-4}\;s^{-2},\;\vartheta_{2}=31.52\;\mathrm{Pa}\cdot s$, $m_{2}=0.05860\;s^{-1}$, and $q_{2}=1.07284\times 10^{-3}\;s^{-2}$. By the formula H(v)=ℋ(1/v), the respective spectrum of relaxation frequencies is as follows:

(65) $$H\left(v\right)=\left[\vartheta_{1}e^{-{\left(v-m_{1}\right)}^{2}/q_{1}}+\vartheta_{2}e^{-{\left(v-m_{2}\right)}^{2}/q_{2}}\right]v,$$

the corresponding real relaxation modulus G(t) is described by Equation (64). 

For experiment, the time interval T=[0,750] seconds is selected in view of the course of the modulus and the Formula (22) important for numerical computations. 

The optimal unconstrained models ${\tilde{H}}_{K}\left(v,\beta_{opt}\right)$ (62) with the parameter vectors ${\tilde{g}}_{K}$ and time-scaling factors $\beta_{opt}$ were determined using the two-level identification scheme proposed in [12] for K=6,7,…,21, and $\beta_{opt}$, regularization parameters $\lambda_{GCV}\left(\beta_{opt}\right)$, norms $\|{{\tilde{g}}_{K}\|}_{2}$ and mean square identification index $Q_{N}\left({\tilde{g}}_{K}\right)/N$ were enclosed in Table 4. The vectors ${\tilde{g}}_{K}$ are given in Table A3 in Appendix B for selected K, from which it can be seen that the number of negative elements is less than for Example 1. The courses of the unconstrained models ${\tilde{H}}_{K}\left(v,\beta_{opt}\right)$ (62) are illustrated by Figure 7, where the real spectrum H(v) (65) and non-negative model ${\hat{H}}_{K}\left(v,\beta_{opt}\right)$ (54) are also given for even K from 6 to 20. For some K the spectra ${\tilde{H}}_{K}\left(v,\beta_{opt}\right)$ (62) are negative for some ranges of the relaxation frequencies, see Figure 7b–d for K=8,10,12. For K=6, the number of model elements is too small to describe the real bimodal spectrum. Only the stronger maximum of the real spectrum is approximated by both models; however, the approximation is more accurate for the non-constrained model, similarly to the approximation of the relaxation modulus measured by identification index. For K=8,10,12, the non-constrained optimal spectrum is negative for some relaxation frequencies, thus applicability of the proposed scheme is necessary to obtain the non-negative model. However, model ${\hat{H}}_{K}\left(v,\beta_{opt}\right)$ is unimodal. For K≥14 the non-constrained spectrum ${\tilde{H}}_{K}\left(v,\beta_{opt}\right)$ is non-negative, becomes bimodal and better approximates both the real relaxation modulus and relaxation spectrum than the model ${\hat{H}}_{K}\left(v,\beta_{opt}\right)$, being still unimodal. 

**Figure 8:**
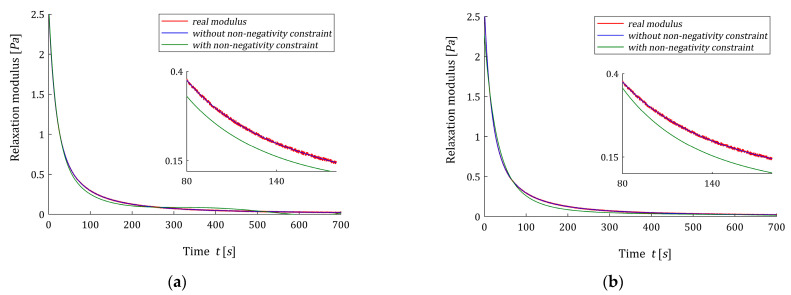
The measurements $\bar{G}\left(t_{i}\right)$ of the real relaxation modulus G(t) (64) (red points) from Example 2 and the optimal approximated models determined without non-negativity constraint ${\tilde{G}}_{K}\left(t,\beta_{opt}\right)$ and with non-negativity constraint ${\hat{G}}_{K}\left(t,\beta_{opt}\right)$ for K model summands: (**a**) K=12; (**b**) K=20.

The non-negative optimal vectors ${\hat{g}}_{K}$ are given in Table A3. For this model, only two to five elements of these vectors are zero. Other numerical data for optimal non-negative models ${\hat{H}}_{K}\left(v,\beta_{opt}\right)$, i.e., norm $\|{{\hat{g}}_{K}\|}_{2}$, index $Q_{N}\left({\hat{g}}_{K}\right)/N$ and the Lagrange multiplier $\hat{\gamma }$ are given in the last columns of Table 4. In Figure 8, the models of the relaxation modulus ${\tilde{G}}_{K}\left(t,\beta_{opt}\right)$ and ${\hat{G}}_{K}\left(t,\beta_{opt}\right)$ corresponding to ${\tilde{H}}_{K}\left(v,\beta_{opt}\right)$ (62) and ${\hat{H}}_{K}\left(v,\beta_{opt}\right)$ (54), respectively, computed according to Equation (12) are plotted for K=12,20, also the measurements $\bar{G}\left(t_{i}\right)$ of the real modulus G(t) (64) are given. 

**Figure 9:**
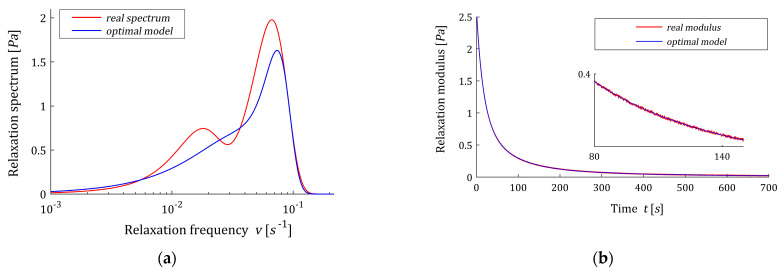
Relaxation frequency spectrum H(v) (65) from Example 2 and the corresponding optimal models of: (**a**) relaxation spectrum ${\tilde{H}}_{K}\left(v,\beta_{opt}\right)={\hat{H}}_{K}\left(v,\beta_{opt}\right)$ and (**b**) relaxation modulus ${\tilde{G}}_{K}\left(t,\beta_{opt}\right)={\hat{G}}_{K}\left(t,\beta_{opt}\right)$ for K=21 summands of the model.

For K=21, vector ${\tilde{g}}_{K}$ is non-negative, thus ${\hat{g}}_{K}={\tilde{g}}_{K}$ also solves the optimization task (27), (28). The related spectrum model ${\hat{H}}_{K}\left(v,\beta_{opt}\right)={\tilde{H}}_{K}\left(v,\beta_{opt}\right)$ is plotted in Figure 9a, while the relaxation modulus model ${\hat{G}}_{K}\left(t,\beta_{opt}\right)$ corresponding to ${\hat{H}}_{K}\left(v,\beta_{opt}\right)$ (54) is given in Figure 9b. The perfect approximation of the relaxation modulus does not match the good approximation of the relaxation spectrum, and the model has also lost its bimodal character. The model already has too many non-zero terms; exactly 21.

However, for K≤20, as K increases, the models ${\tilde{H}}_{K}\left(v,\beta_{opt}\right)$ (62) determined without non-negativity constraint approximates the bimodal spectrum more and more closely, the model determined with the non-negativity constraint does not. Models ${\hat{H}}_{K}\left(v,\beta_{opt}\right)$ (54), with increasing K, better and better approximate the second, major, maximum, but it does not model the first, minor, maximum even for 20 components.

#### 3.10.3. Example 3

Now, we consider viscoelastic material of unimodal relaxation spectrum described by distribution:

(66) $$H\left(v\right)=\vartheta ve^{-{\left(v-m\right)}^{2}/q}.$$

where the parameters are as follows: ϑ=39 Pa·s, $m=0.045\;s^{-1}$, and $q=1.173\times 10^{-3}\;s^{-2}$. The corresponding real relaxation modulus G(t) is described by one component of the model of the form (64). In experiment the time interval T=[0,500] seconds was applied, which resulted from the inspection of the course of G(t). 

For K=3,4,…,8, the optimal time-scaling factors $\beta_{opt}$, the related regularization parameters $\lambda_{GCV}$, the mean optimal identification indices $Q_{N}\left({\tilde{g}}_{K}\right)/N$ and square norms $\|{{\tilde{g}}_{K}\|}_{2}$ are given in Table 5. The vectors of optimal model parameters ${\tilde{g}}_{K}$ are given in Table A4 in Appendix B; the elements of these vectors are both negative and positive. related to model ${\tilde{H}}_{K}\left(v,\beta \right)$ (62) 

**Figure 10:**
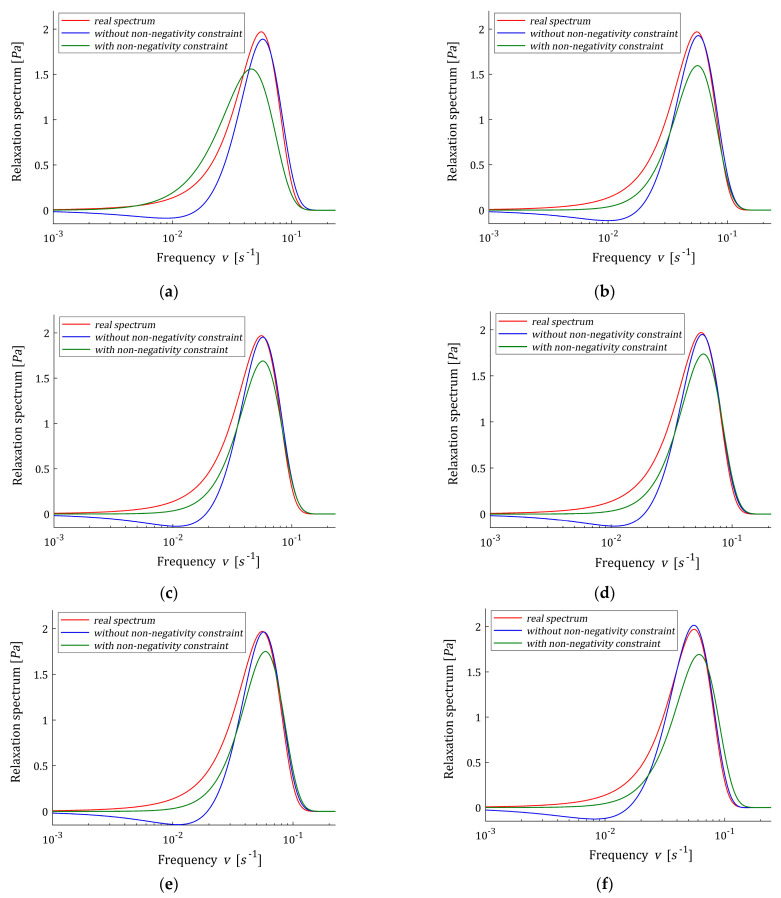
Relaxation spectrum H(v) (66) (solid red line) from Example 3 and the corresponding models: without non-negativity constraint ${\tilde{H}}_{K}\left(v,\beta_{opt}\right)$ (62) and with non-negativity constraint ${\hat{H}}_{K}\left(v,\beta_{opt}\right)$ (54) for K summands of the model: (**a**) K=3; (**b**) K=4; (**c**) K=5; (**d**) K=6; (**e**) K=7; (**f**) K=8.

**Figure 11:**
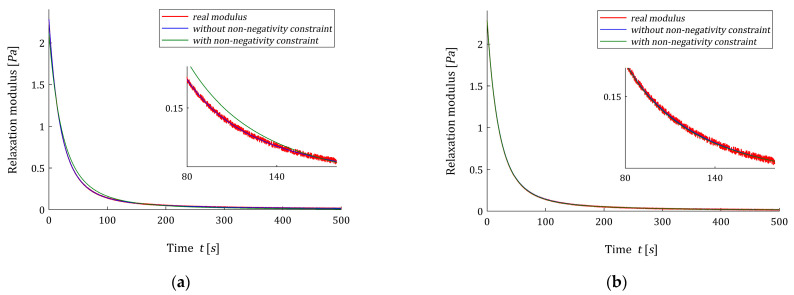
The measurements $\bar{G}\left(t_{i}\right)$ (red points) of the real relaxation modulus G(t) from Example 3 and the models of the relaxation modulus corresponding to the optimal spectra models determined without non-negativity constraint ${\tilde{H}}_{K}\left(v,\beta_{opt}\right)$ (62) (blue line) and with non-negativity constraint ${\hat{H}}_{K}\left(v,\beta_{opt}\right)$ (54) (green line) for: (**a**) K=3; (**b**) K=7 summands of the model.

Next, for time-scale factor $\beta =\beta_{opt}$ the optimal non-negative models ${\hat{H}}_{K}\left(v,\beta \right)$ (54) were determined; the smoothing parameter κ was selected several times until a satisfactory accuracy of the fit of the model to the experimental data was obtained. The non-negative optimal parameters ${\hat{g}}_{K}$ (50) are given in Table A4, while the multiplies $\hat{\gamma }$ defined in Equation (30), norms $\|{\hat{g}\|}_{2}$, and optimal identification indices $Q_{N}\left({\hat{g}}_{K}\right)/N$ are given in Table 5. For K=3,4,…12, the real spectrum H(v) (66), optimal models ${\tilde{H}}_{K}\left(v,\beta_{opt}\right)$ (62) and ${\hat{H}}_{K}\left(v,\beta_{opt}\right)$ (54) are plotted in Figure 10. The norms $\|{\hat{g}\|}_{2}$ are equal to the smoothing parameters κ, which are assumed smaller than the norms $\|{{\tilde{g}}_{K}\|}_{2}$, since a quick inspection of the data from Table A4 shows that many elements of the vector ${\tilde{g}}_{K}$ are negative (even, six by eight for K=8). In Figure 11 the optimal models of the relaxation modulus $G_{K}\left(t\right)$ related to unconstrained ${\tilde{H}}_{K}\left(v,\beta_{opt}\right)$ (62) and non-negative ${\hat{H}}_{K}\left(v,\beta_{opt}\right)$ (54) relaxation spectra are plotted for K=3 and K=7. 

Figure 10 shows that the ${\tilde{H}}_{K}\left(v,\beta_{opt}\right)$ model, determined without additional non-negativity constraint, is negative in some range of frequencies for any K. However, the identification index $Q_{N}\left({\hat{g}}_{K}\right)/N$ is from 1.08 (for K=7) to 68.1 (for K=3) times greater than $Q_{N}\left({\tilde{g}}_{K}\right)/N$ obtained for unconstrained model (Table 5), the inspection of Figure 10 shows that model ${\hat{H}}_{K}\left(v,\beta_{opt}\right)$ well approximates the real spectrum, and the quality of this approximation improves with increasing K. For K=3,…,8, two elements of the vector ${\hat{g}}_{K}$ are zero, i.e., for K=3 only one element of the vector ${\hat{g}}_{K}$ is non-zero and in result index $Q_{N}\left({\hat{g}}_{K}\right)/N$ is the biggest, see also Figure 11a. Analysis of both the values of identification index $Q_{N}\left({\hat{g}}_{K}\right)/N$ and Figure 10 indicates that the best model with non-negativity constraint was obtained for K=7. For K=7, both the relaxation modulus models practically coincide with the measurement points and with each other, see Figure 11b. Increasing the number of model components to K=8 no longer corrects the model. 

#### 3.10.4. Discussion

In Example 1, the peaks of the spectrum are more distant than in Example 2. For successive k, the maxima of the basis functions $h_{k}\left(\tau ,\alpha \right)$ (3) are more distant than the maxima of the functions $h_{k}\left(v,\beta \right)$ (11). Thus, the relaxation time model ${ℋ}_{K}\left(\tau ,\alpha \right)$ (5) was more appropriate for modeling spectrum in Example 1, than model $H_{K}\left(v,\beta \right)$ (10). For the same reason, in Example 1, it was enough to use K=12 model components, while in Example 2, many more model components were necessary (K=21) to obtain a satisfactory approximation of the real relaxation modulus and spectrum.

The parameter vectors ${\hat{g}}_{K}$ of the models determined with non-negativity constraint have zero elements. Therefore, these models are composed of fewer items than index K would indicate. However, the model of full dimension K must be determined on the identification stage. The proposed approach, effective for the unimodal spectrum, is less effective for the multi-modal spectra, because the additional non-negativity constraint reduces the set of admissible models and, therefore, makes it impossible to achieve such a good fit of the model to the experiment data as for the model determined without this constraint.

Additionally, the examples showed that a new model of the frequency spectrum can be applied for unimodal and bimodal spectra approximation when the regularized least-squares identification with optimal choice of the time-scale factor is used without additional non-negativity constraint. 

### 3.11. Applicability of the Scheme to Discrete Relaxation Spectra Identification

Assume, as above, that the experiment resulted in a set of the measurements $\left\{\bar{G}\left(t_{i}\right)=G\left(t_{i}\right)+z\left(t_{i}\right)\right\}$ at the times $t_{i}\geq 0$, i=1,…,N. By (1), for any time $t_{i}$ we have:

(67) $$G\left(t_{i}\right)=\int_{0}^{\infty }\frac{ℋ\left(\tau \right)}{\tau }e^{-t_{i}/\tau }d\tau .$$

Let $\tau_{k}=\frac{\Delta \tau }{2}+k\Delta \tau$, where k=0,1,…,K−1 and Δτ>0 is the length of integration step. Then, for any i=1,…,N, the integral of the right-hand side of Equation (67) can be approximated by:

(68) $$G\left(t_{i}\right)≅\sum_{k=0}^{K-1}\frac{ℋ\left(\tau_{k}\right)}{\tau_{k}}e^{-t_{i}/\tau_{k}}\Delta \tau ,$$

whenever the number of subintervals K and the integration step Δτ are such that the integrand is sufficiently small for $\tau \geq \left(K-\frac{1}{2}\right)\Delta \tau$. Denoting:

(69) $$g_{K}=\left[\begin{array}{c} ℋ\left(\tau_{0}\right)\\ \vdots \\ ℋ\left(\tau_{K-1}\right) \end{array}\right],\;\Phi_{N,K}=\left[\begin{array}{ccc} \frac{\Delta \tau }{\tau_{0}}e^{-t_{1}/\tau_{0}} & \cdots & \frac{\Delta \tau }{\tau_{K-1}}e^{-t_{1}/\tau_{K-1}}\\ \vdots & \ddots & \vdots \\ \frac{\Delta \tau }{\tau_{0}}e^{-t_{N}/\tau_{0}} & \cdots & \frac{\Delta \tau }{\tau_{K-1}}e^{-t_{N}/\tau_{K-1}} \end{array}\right],$$

compare Equation (26), the set of discretized model equations takes the form:

(70) $$G_{M}≅\Phi_{N,K}g_{K},$$

with vector $g_{K}$ of unknown relaxation spectrum at relaxation times $\tau_{k}$ and known elements of the matrix $\Phi_{N,K}$, where $G_{M}$ is the vector of the relaxation modulus of model (1) at times $t_{i}$, defined by analogy to the vector of relaxation modulus measurements ${\bar{G}}_{N}$. Now, the square of the model (70) error is described by identification index $Q_{N}\left(g_{K}\right)$ (25) and the proposed identification scheme can be applied for determining the best nonnegative approximations of the discretized relaxation time spectrum. As a result, the set of pairs $\left(\tau_{k},\;\hat{ℋ}\left(\tau_{k}\right)\right)$, for k=0,1,…,K−1, where the optimal $\hat{ℋ}\left(\tau_{k}\right)$ are uniquely given by the optimal model parameter ${\hat{g}}_{K}$ according to Equation (69). The approximation of the the discrete spectrum becomes more accurate as more rectangles are used in the series (68). By analogous discretization of Equation (2), discrete relaxation frequency spectrum can be determined. 

The simple rectangular (midpoint) rule with equally spaced points $\tau_{k}$ is applied here; however, other, more sophisticated quadratures can be also used.

## 4. Conclusions

In this paper, a new hierarchical identification scheme for recovery of the non-negative continuous relaxation spectra has been derived. The scheme can be applied to identify both relaxation time and frequency spectra using the relaxation test data. Two classes of models are considered; both are based on an expansion of an unknown spectrum into a series of non-negative basis functions. The continuous spectrum of relaxation times was approximated by finite series of power-exponential basis functions, with the components of the relaxation modulus model described by the product of power of time and the modified Bessel function of the second kind. For modeling of the relaxation frequency spectrum, the basis functions described by the product of power of time and square exponential functions were applied. The components of the related relaxation modulus model were proven to be described by compact recurrence formulas expressed in terms of the products of power of time, exponential, and complementary error functions. The quadratic identification index related to the relaxation modulus measurements was used, and an additional smoothing constraint was imposed on the model parameters to ensure the problem was well-posed. The numerical experiments showed that both considered classes of models can be applied for unimodal and bimodal relaxation spectra modeling with additional non-negativity constraints. The model of the relaxation time spectrum using the modified Bessel functions can be recommended for modeling bimodal spectra with the peaks more distant than the relaxation frequency model.

However, the examples showed that in many cases, the non-negative models of the relaxation spectra or models non-negative for almost all arguments can be obtained also using the classical approach, without the additional constraint of the model parameters non-negativity, whenever the basis functions of the relaxation spectrum model are non-negatively defined. Thus, the following procedure can be recommended for the non-negative relaxation spectrum determination. First, find the best model of the relaxation spectrum using regularized least-squares identification and check the definiteness of the designated model. If this model is non-negative over a significant range of relaxation times or frequencies, accept it. Otherwise, apply the proposed two-stage hierarchical algorithm and determine the nonnegative relaxation spectrum model. 

However, the best non-negative model can be obtained by solving the original infinite-dimensional task of optimal approximation of the real spectrum in the class of continuous non-negative functions by applying the calculus of variations technique. It will be the subject of further work. 

## Figures and Tables

**Table 1:** Ranges of the applicability of the models (10) and (12) for various time-scale parameters for K=5 and K=12.

**Time-Scale Factor** β $\left[s^{2}\right]$	K=5	K=5	K=12	K=12
	$\mathrm{Range}{\;}^{1}\;\mathrm{of}\;\mathrm{Relaxation}\;\mathrm{Frequencies}\;v_{app}\left(\beta \right)$ $\left[s^{-1}\right]$	$\mathrm{Range}{\;}^{1}\;\mathrm{of}\;\mathrm{Times}\;t_{app}\left(\beta \right)$ [s]	$\mathrm{Range}{\;}^{1}\;\mathrm{of}\;\mathrm{Relaxation}\;\mathrm{Frequencies}\;v_{app}\left(\beta \right)$ $\left[s^{-1}\right]$	$\mathrm{Range}{\;}^{1}\;\mathrm{of}\;\mathrm{Times}\;t_{app}\left(\beta \right)$ [s]
0.0000001	10,791.76	0.006284	13,355.44	0.006284
0.000001	3412.50	0.01987	4223.70	0.01987
0.00001	1079.55	0.0628	1335.50	0.0628
0.0001	341.40	0.199	422.53	0.199
0.001	108.17	0.628	133.80	0.628
0.01	34.25	1.987	42.32	1.987
0.1	10.83	6.284	13.40	6.284
1	3.45	19.872	4.23	19.872
10	1.08	62.870	1.35	62.870
100	0.345	198.57	0.425	198.57

^1^ The upper bounds $t_{app}\left(\beta \right)$ (23) and $v_{app}\left(\beta \right)$ (24) of the applicability intervals $\left[0,t_{app}\left(\beta \right)\right]$ and $\left[0,v_{app}\left(\beta \right)\right]$ are given.

**Table 2:** The square roots of the largest ${\bar{\sigma }}_{1}\left(\Theta \right)$ and minimal ${\bar{\sigma }}_{min}\left(\Theta \right)$ singular values of the matrix Θ (56)–(58) for K=4,5, …12 model summands.

K	4	5	6	7	8	9	10	11	12
$\sqrt{{\bar{\sigma }}_{1}\left(\Theta \right)}$	2.680365	2.946643	3.176727	3.380015	3.562632	3.728808	3.881584	4.023221	4.155442
$\sqrt{{\bar{\sigma }}_{min}\left(\Theta \right)}$	0.031663	0.008960	0.002528	7.110 × 10^−4^	1.992 × 10^−4^	5.565 × 10^−5^	1.550 × 10^−5^	4.306 × 10^−6^	4.306 × 10^−6^

**Table 3:** The parameters of the optimal models in Example 1 for the models ${\tilde{ℋ}}_{K}\left(\tau ,\alpha_{opt}\right)$ (61) without non-negativity constraint: optimal time-scale factors $\alpha_{opt}$, the mean quadratic identification indices $Q_{N}\left({\tilde{g}}_{K}\right)/N$ (c.f., definition (25)) and the square norms $\|{{\tilde{g}}_{K}\|}_{2}$ for the optimal model parameters ${\tilde{g}}_{K}$ [12] (Table A3) and for optimal models ${\hat{ℋ}}_{K}\left(\tau ,\alpha_{opt}\right)$ (54) determined with the non-negativity constraint: the multiplier $\hat{\gamma }$ defined by the optimization task (30) and the norms $\|{{\hat{g}}_{K}\|}_{2}$ (equal to smoothing parameters κ) and identification indices $Q_{N}\left({\hat{g}}_{K}\right)/N$ corresponding to non-negative optimal parameters ${\hat{g}}_{K}$ (50) from Table A2.

Without Non-Negativity Constraint				With Non-Negativity Constraint		
K	$\alpha_{opt}$ $\left[s^{-1}\right]$	$Q_{N}\left({\tilde{g}}_{K}\right)/N$ $\left[Pa^{2}\right]$	$\|{{\tilde{g}}_{K}\|}_{2}$ [Pa]	$\hat{\gamma }$	$Q_{N}\left({\hat{g}}_{K}\right)/N$ $\left[Pa^{2}\right]$	$\|{{\hat{g}}_{K}\|}_{2}=\kappa$ [Pa]
3	0.00520	8.63505 × 10^−4^	0.7055	2.677 × 10^−3^	8.64732 × 10^−4^	0.7
4	0.01675	3.43945 × 10^−5^	7.3485	2.88 × 10^−6^	1.99487 × 10^−3^	1.1644
5	0.02025	2.71552 × 10^−5^	4.6724	7.88 × 10^−6^	1.78025 × 10^−3^	1.1394
6	0.02375	2.48511 × 10^−5^	3.6493	1.66 × 10^−3^	1.53113 × 10^−3^	1.1749
7	0.02655	2.48256 × 10^−5^	2.8846	1.61	1.18092 × 10^−3^	1.2169
8	0.02865	2.51617 × 10^−5^	2.3555	7.48 × 10^−6^	8.136760 × 10^−4^	1.2634
9	0.03005	2.52412 × 10^−5^	2.0639	8.87	4.90203 × 10^−4^	1.3110
10	0.03215	2.51143 × 10^−5^	1.9058	7.42	3.06912 × 10^−4^	1.3872
11	0.03390	2.48521 × 10^−5^	1.8020	4.79 × 10^−7^	1.77760 × 10^−4^	1.4485
12	0.03670	2.44452 × 10^−5^	1.7198	1.739 × 10^−3^	1.45878 × 10^−4^	1.5083

**Table 4:** The parameters of the optimal models in Example 2 for the models ${\tilde{H}}_{K}\left(v,\beta_{opt}\right)$ (62) determined without non-negativity constraint: optimal time-scale factors $\beta_{opt}$, regularization parameters $\lambda_{GCV}\left(\beta_{opt}\right)$ (for details see [12]), the mean quadratic identification indices $Q_{N}\left({\tilde{g}}_{K}\right)/N$ (compare (25)) and the norms $\|{{\tilde{g}}_{K}\|}_{2}$ for the optimal model parameters ${\tilde{g}}_{K}$ given in Table A3 and for optimal models ${\hat{H}}_{K}\left(v,\beta_{opt}\right)$ (54) determined with the non-negativity constraint: the multiplier $\hat{\gamma }$ defined by the optimization task (30) and the norms $\|{{\hat{g}}_{K}\|}_{2}$ (equal to smoothing parameters κ) and identification indices $Q_{N}\left({\hat{g}}_{K}\right)/N$ corresponding to non-negative optimal parameters ${\hat{g}}_{K}$ (50), see Table A3.

Without Non-Negativity Constraint				With Non-Negativity Constraint			
K	$\beta_{opt}$ $\left[s^{2}\right]$	$\lambda_{GCV}\left(\beta_{opt}\right)$	$Q_{N}\left({\tilde{g}}_{K}\right)/N\;$ $\left[Pa^{2}\right]$	$\|{{\tilde{g}}_{K}\|}_{2}$ [Pa]	$\hat{\gamma }$	$Q_{N}\left({\hat{g}}_{K}\right)/N\;$ $\left[Pa^{2}\right]$	$\|{{\hat{g}}_{K}\|}_{2}$ [Pa]
6	392	1.7651 × 10^−6^	8.40211 × 10^−6^	2.814225	7.7539× 10^−5^	3.09039 × 10^−5^	1.23554
8	496	7.4141 × 10^−6^	8.25509 × 10^−6^	4.161575	0.0269	5.89873 × 10^−5^	1.65147
10	610	2.5071 × 10^−6^	8.24771 × 10^−6^	6.084721	9.31× 10^−7^	2.14951× 10^−4^	0.983014
12	769	2.1041 × 10^−7^	8.24645 × 10^−6^	14.724716	1.49× 10^−4^	7.55925 × 10^−4^	1.17871
14	684	9.010 × 10^−5^	8.25172× 10^−6^	1.266785	7.88× 10^−4^	1.60128 × 10^−4^	1.42615
15	809	2.390 × 10^−5^	8.24382 × 10^−6^	1.762488	0.5564	4.02679 × 10^−4^	1.54932
16	777	1.010 × 10^−5^	8.24388 × 10^−6^	1.576049	0.5288	1.77827 × 10^−4^	1.28109
17	1004	1.6491 × 10^−6^	8.24399 × 10^−6^	2.605599	0.0976	2.9148 × 10^−4^	1.169359
18	860	1.530 × 10^−4^	8.24674 × 10^−6^	0.975532	0.3973	8.049 × 10^−5^	0.82328
19	1168.5	3.600 × 10^−6^	8.24483 × 10^−6^	1.861569	0.7793	6.4736 × 10^−4^	1.16471
20	1150.75	4.530 × 10^−5^	8.31429 × 10^−6^	1.071904	0.9573	8.4609× 10^−4^	1.07082
21	1084.25	1.4140 × 10^−4^	9.79755 × 10^−6^	0.726038	0.000115	9.79755 × 10^−6^	0.726038

**Table 5:** The parameters of the optimal models in Example 3 for the model ${\tilde{H}}_{K}\left(v,\beta_{opt}\right)$ (62) without non-negativity constraint: optimal time-scale factors $\beta_{opt}$, regularization parameters $\lambda_{GCV}$ [12], the mean quadratic identification indices $Q_{N}\left({\tilde{g}}_{K}\right)/N$ and the square norms $\|{{\tilde{g}}_{K}\|}_{2}$ for the optimal model parameters ${\tilde{g}}_{K}$ [12] and for optimal model ${\hat{H}}_{K}\left(v,\beta_{opt}\right)$ (54) determined with the non-negativity constraint: the multiplier $\hat{\gamma }$ defined in Equation (30) and the norms $\|{{\hat{g}}_{K}\|}_{2}$ (equal to smoothing parameters κ) and identification indices $Q_{N}\left({\hat{g}}_{K}\right)/N$ corresponding non-negative optimal parameters ${\hat{g}}_{K}$ (50).

Without Non-Negativity Constraint				With Non-Negativity Constraint			
K	$\beta_{opt}$ $\left[s^{2}\right]$	$\lambda_{GCV}$	$Q_{N}\left({\tilde{g}}_{K}\right)/N$ $\left[Pa^{2}\right]$	$\|{{\tilde{g}}_{K}\|}_{2}$ [Pa]	$\hat{\gamma }$	$Q_{N}\left({\hat{g}}_{K}\right)/N$ $\left[Pa^{2}\right]$	$\|{{\hat{g}}_{K}\|}_{2}=\kappa$ [Pa]
3	477	4.0 × 10^−6^	8.35568 × 10^−6^	1.9731	1.86 × 10^−5^	5.69022 × 10^−4^	1.5606
4	495	3.9 × 10^−6^	8.26117 × 10^−6^	2.1789	5.277 × 10^−3^	5.15926 × 10^−4^	1.4038
5	507	7.7 × 10^−6^	8.25179 × 10^−6^	2.3219	4.161 × 10^−3^	1.00861 × 10^−4^	1.3122
6	517	4.9 × 10^−6^	8.25048 × 10^−6^	2.4096	6.11 × 10^−6^	2.12842 × 10^−5^	1.2277
7	511	1.9 × 10^−6^	8.25042 × 10^−6^	2.3902	2.63 × 10^−4^	8.87258 × 10^−6^	1.1912
8	416	2.3 × 10^−6^	8.24898 × 10^−6^	2.4891	1.942 × 10^−3^	1.41488 × 10^−5^	1.2510

## Data Availability

Not applicable.

## Appendix A

### Appendix A.1. Proof of Theorem 2

First we prove (15) and (16) for k=0 and k=1. To derive (15), in view of (11) and (13), we have

$$\phi_{0}\left(t,\beta \right)=\sqrt{2\beta e}\int_{0}^{\infty }e^{-\beta v^{2}}e^{-tv}dv,$$

whence, by applying the substitution $u=\sqrt{\beta }v+t/2\sqrt{\beta }$, we obtain:

$$\phi_{0}\left(t,\beta \right)=\sqrt{2e}\cdot e^{\frac{t^{2}}{4\beta }}\;\int_{t/2\sqrt{\beta }}^{\infty }\;e^{-u^{2}}du,$$

which, having in mind the definition (17) of the complementary error function, can be rewritten as (15).

For k≥0, on the basis of (11) and (13), the following general differential property holds

(A1) $$\phi_{k+1}\left(t,\beta \right)=\left(-1\right)\sqrt{\frac{2\beta e}{k+2}}{\left(\frac{k+1}{k+2}\right)}^{\frac{k+1}{2}}\frac{\partial \phi_{k}\left(t,\beta \right)}{\partial t}.$$

Whence, putting k=0, the integral $\phi_{1}\left(t,\beta \right)$ can be expressed as

(A2) $$\phi_{1}\left(t,\beta \right)=\left(-1\right)\sqrt{\frac{\beta e}{2}}\cdot \frac{\partial \phi_{0}\left(t,\beta \right)}{\partial t}.$$

Since, in view of (17),

(A3) $$\frac{derfc\left(x\right)}{dx}=\frac{-2}{\sqrt{\pi \;}\;}e^{-x^{2}},$$

bearing in mind (15), Equation (A2) can be rewritten as

$$\phi_{1}\left(t,\beta \right)=\left(-1\right)\sqrt{\frac{\beta e}{2}}\cdot \sqrt{\frac{\pi e}{2}}\;\left[\frac{t}{2\beta }e^{\frac{t^{2}}{4\beta }}erfc\left(\frac{t}{2\sqrt{\beta }}\right)-\frac{2}{\sqrt{\pi \;}\;}\frac{1}{2\sqrt{\beta }}e^{\frac{t^{2}}{4\beta }}e^{\frac{-t^{2}}{4\beta }}\right],$$

whereas the next formula follows

(A4) $$\phi_{1}\left(t,\beta \right)=\frac{e}{2}\;\left[1-\frac{1}{\sqrt{2\beta e}}\sqrt{\frac{\pi e}{2}}t\;e^{\frac{t^{2}}{4\beta }}erfc\left(\frac{t}{2\sqrt{\beta }}\right)\right],$$

which, in view of (15), can be expressed as (16).

For the proof of recurrence formula (14) mathematical induction will be used. In the base case of the proof by induction, for k=1, by (A1), we have

$$\phi_{2}\left(t,\beta \right)=\left(-1\right)\sqrt{\frac{2\beta e}{3}}\left(\frac{2}{3}\right)\frac{\partial \phi_{1}\left(t,\beta \right)}{\partial t},$$

which, combined with (16) yields

$$\phi_{2}\left(t,\beta \right)=\sqrt{\frac{2\beta e}{3}}\left(\frac{2}{3}\right)\frac{e}{2}\frac{1}{\sqrt{2\beta e}}\left[\;\phi_{0}\left(t,\beta \right)+t\frac{\partial \phi_{0}\left(t,\beta \right)}{\partial t}\right].$$

Whence, including (A2), we obtain formula (14) for k=1, i.e.,

$$\phi_{2}\left(t,\beta \right)=e{\left(\frac{1}{3}\right)}^{\frac{3}{2}}\left[\;\phi_{0}\left(t,\beta \right)-2\frac{1}{\sqrt{2\beta e}}t\phi_{1}\left(t,\beta \right)\right].$$

Now, in the induction step, let us assume that (14) holds for k≥1. We prove that it holds also for k+1. For k+1, differential formula (A1) yields

(A5) $$\phi_{k+2}\left(t,\beta \right)=\left(-1\right)\sqrt{\frac{2\beta e}{k+3}}{\left(\frac{k+2}{k+3}\right)}^{\frac{k+2}{2}}\frac{\partial \phi_{k+1}\left(t,\beta \right)}{\partial t}.$$

By the induction hypothesis, from (14) we immediately have

(A6) $$\frac{\partial \phi_{k+1}\left(t,\beta \right)}{\partial t}=e\;{\left(\frac{k}{k+2}\right)}^{\frac{k+2}{2}}\psi_{k}\left(t,\beta \right),$$

where

(A7) $$\psi_{k}\left(t,\beta \right)=\frac{\partial \phi_{k-1}\left(t,\beta \right)}{\partial t}-\frac{1}{\sqrt{2\beta e}\;\sqrt{k}}{\left(\frac{k+1}{k}\right)}^{\frac{k+1}{2}}\phi_{k}\left(t,\beta \right)-\frac{1}{\sqrt{2\beta e}\;\sqrt{k}}{\left(\frac{k+1}{k}\right)}^{\frac{k+1}{2}}t\frac{\partial \phi_{k}\left(t,\beta \right)}{\partial t}.$$

Taking into account (A1), derivatives $\frac{\partial \phi_{k-1}\left(t,\beta \right)}{\partial t}$ and $\frac{\partial \phi_{k}\left(t,\beta \right)}{\partial t}$ can be expressed as the functions of $\phi_{k}\left(t,\beta \right)$ and $\phi_{k+1}\left(t,\beta \right)$, respectively. Whence, function $\psi_{k}\left(t,\beta \right)$ (A7), after algebraic manipulations, can be expressed as

(A8) $$\psi_{k}\left(t,\beta \right)=\left(-1\right)\frac{1}{\sqrt{2\beta e}\;}\frac{k+1}{\;\sqrt{k}}{\left(\frac{k+1}{k}\right)}^{\frac{k+1}{2}}\phi_{k}\left(t,\beta \right)+\frac{1}{2\beta e}{\left(\frac{k+2}{k}\right)}^{\frac{k+2}{2}}t\phi_{k+1}\left(t,\beta \right).$$

Substituting $\psi_{k}\left(t,\beta \right)$ (A8) into (A6) results in

$$\frac{\partial \phi_{k+1}\left(t,\beta \right)}{\partial t}=-e\;{\left(\frac{k}{k+2}\right)}^{\frac{k+2}{2}}\left[\frac{1}{\sqrt{2\beta e}\;}\frac{k+1}{\;\sqrt{k}}{\left(\frac{k+1}{k}\right)}^{\frac{k+1}{2}}\phi_{k}\left(t,\beta \right)-\frac{1}{2\beta e}{\left(\frac{k+2}{k}\right)}^{\frac{k+2}{2}}t\phi_{k+1}\left(t,\beta \right)\right],$$

which, combined with (A5), yields

$$\phi_{k+2}\left(t,\beta \right)=e\;\sqrt{\frac{2\beta e}{k+3}}{\left(\frac{k}{k+3}\right)}^{\frac{k+2}{2}}\left[\frac{1}{\sqrt{2\beta e}\;}\frac{k+1}{\;\sqrt{k}}{\left(\frac{k+1}{k}\right)}^{\frac{k+1}{2}}\phi_{k}\left(t,\beta \right)-\;\frac{1}{2\beta e}{\left(\frac{k+2}{k}\right)}^{\frac{k+2}{2}}t\phi_{k+1}\left(t,\beta \right)\right],$$

whence, after algebraic manipulations, we finally obtain

$$\phi_{k+2}\left(t,\beta \right)=e\;{\left(\frac{k+1}{k+3}\right)}^{\frac{k+2}{2}}\left[\phi_{k}\left(t,\beta \right)-\frac{1}{\sqrt{2\beta e}}\frac{1}{\sqrt{k+1}}{\left(\frac{k+2}{k+1}\right)}^{\frac{k+2}{2}}t\phi_{k+1}\left(t,\beta \right)\right].$$

Thus, theorem is proved. □

### Appendix A.2. Proof of Theorem 3

Mathematical induction will be used again. In the base step, we prove (18), (20) for k=0 and (18), (19) for k=1 and k=2. The function $\phi_{0}\left(t,\beta \right)$ (15) can be expressed as

$$\phi_{0}\left(t,\beta \right)=\sqrt{\frac{\pi e}{2}}\;\frac{erfc\left(\frac{t}{2\sqrt{\beta }}\right)}{e^{\frac{-t^{2}}{4\beta }}},$$

where both the numerator and denominator tends to zero, when t → ∞. Thus, using the L’Hospital’s rule, having in mind (A3), we obtain

(A9) $$\underset{t\to \infty \;}{lim\;}\phi_{0}\left(t,\beta \right)=\sqrt{\frac{\pi e}{2}}\;\;\underset{t\to \infty \;}{lim\;}\frac{-\frac{2}{\sqrt{\pi \;}\;}\;\frac{1}{2\sqrt{\beta }}e^{\frac{-t^{2}}{4\beta }}}{-\frac{t}{2\beta }e^{\frac{-t^{2}}{4\beta }}}=\sqrt{\frac{\pi e}{2}}\;\;\underset{t\to \infty \;}{lim\;}\frac{\frac{1}{\sqrt{\pi \beta \;}\;}\;}{\frac{t}{2\beta }}=0.$$

To prove (20), the product $t\phi_{0}\left(t,\beta \right)$ is rewritten as

$$t\phi_{0}\left(t,\beta \right)=\sqrt{\frac{\pi e}{2}}\;\frac{erfc\left(\frac{t}{2\sqrt{\beta }}\right)}{\frac{1}{t}\;e^{\frac{-t^{2}}{4\beta }}},$$

whence, by the L’Hospital’s rule we have

(A10) $$\underset{t\to \infty \;}{lim\;}t\phi_{0}\left(t,\beta \right)=\sqrt{\frac{\pi e}{2}}\;\underset{t\to \infty \;}{lim\;}\;\frac{-\frac{2}{\sqrt{\pi \;}\;}\;\frac{1}{2\sqrt{\beta }}e^{\frac{-t^{2}}{4\beta }}}{\frac{1}{t}\;\left(-\frac{t}{2\beta }\right)e^{\frac{-t^{2}}{4\beta }}-\frac{1}{t^{2}}\;e^{\frac{-t^{2}}{4\beta }}}=\sqrt{\frac{\pi e}{2}}\;\underset{t\to \infty \;}{lim\;}\;\frac{\frac{1}{\sqrt{\pi \beta \;}\;}\;}{\;\frac{1}{2\beta }+\frac{1}{t^{2}}\;}=\sqrt{2e\beta \;}.$$

For k=1, by (16) and (A10), we immediately have

$$\underset{t\to \infty \;}{lim\;}\phi_{1}\left(t,\beta \right)=\frac{e}{2}\;\left[1-\frac{1}{\sqrt{2\beta e}}\sqrt{2\beta e}\right]=0.$$

Having in mind (A4), the product $t\phi_{1}\left(t,\beta \right)$ can be expressed as a fraction

$$t\phi_{1}\left(t,\beta \right)=\frac{e}{2}\;\frac{\left[\frac{1}{t}e^{\frac{-t^{2}}{4\beta }}-\frac{1}{\sqrt{2\beta e}}\sqrt{\frac{\pi e}{2}}\;erfc\left(\frac{t}{2\sqrt{\beta }}\right)\right]}{\frac{1}{t^{2}}\;e^{\frac{-t^{2}}{4\beta }}},$$

which numerator and denominator tends to zero, when t → ∞. Thus, the L’Hospital’s rule and (A3) yield

$$\underset{t\to \infty \;}{lim\;}t\phi_{1}\left(t,\beta \right)=\frac{e}{2}\underset{t\to \infty \;}{lim\;}\;\frac{\frac{-1}{t^{2}}e^{\frac{-t^{2}}{4\beta }}-\frac{t}{2\beta }\times \frac{1}{t}e^{\frac{-t^{2}}{4\beta }}+\frac{1}{2\sqrt{\beta }}\frac{2}{\sqrt{\pi \;}\;}\frac{1}{\sqrt{2\beta e}}\sqrt{\frac{\pi e}{2}}\;e^{\frac{-t^{2}}{4\beta }}}{\frac{-2}{t^{3}}\;e^{\frac{-t^{2}}{4\beta }}-\frac{t}{2\beta }\frac{1}{t^{2}}\;e^{\frac{-t^{2}}{4\beta }}},$$

whence the next limit directly follows

(A11) $$\underset{t\to \infty \;}{lim\;}t\phi_{1}\left(t,\beta \right)=\frac{e}{2}\;\underset{t\to \infty \;}{lim\;}\frac{\frac{-1}{t^{2}}-\frac{1}{2\beta }+\frac{1}{2\beta }\;}{\frac{-2}{t^{3}}-\frac{1}{2\beta }\frac{1}{t}}=\frac{e}{2}\;\underset{t\to \infty \;}{lim\;}\frac{1\;}{\frac{2}{t}+\frac{1}{2\beta }t}=0.$$

Similarly, to prove (19) for k=2, the product $t\phi_{2}\left(t,\beta \right)$, due to (14), (16) and (15), can be expressed as

$$t\phi_{2}\left(t,\beta \right)=e\;{\left(\frac{1}{3}\right)}^{\frac{3}{2}}\left[\frac{\sqrt{\frac{\pi e}{2}}\;\;\frac{1}{t^{2}}erfc\left(\frac{t}{2\sqrt{\beta }}\right)-\frac{e}{\sqrt{2\beta e}\;}\;\left[\frac{1}{t}e^{\frac{-t^{2}}{4\beta }}-\frac{1}{\sqrt{2\beta e}}\;\sqrt{\frac{\pi e}{2}}\;\;erfc\left(\frac{t}{2\sqrt{\beta }}\right)\right]}{\frac{1}{t^{3}}e^{\frac{-t^{2}}{4\beta }}}\right],$$

where numerator and denominator tends to zero, when t → ∞. Thus, using again the L’Hospital’s rule and differential formula (A3) we obtain

$$\underset{t\to \infty \;}{lim\;}t\phi_{2}\left(t,\beta \right)=\;e{\left(\frac{1}{3}\right)}^{\frac{3}{2}}\underset{t\to \infty \;}{lim\;}[\frac{-\sqrt{\frac{\pi e}{2}}\;\;\frac{2}{t^{3}}erfc\left(\frac{t}{2\sqrt{\beta }}\right)-\sqrt{\frac{e}{2\beta }}\;\;\frac{1}{t^{2}}e^{\frac{-t^{2}}{4\beta }}-\frac{e}{\sqrt{2\beta e}\;}\;\left[\frac{-1}{t^{2}}e^{\frac{-t^{2}}{4\beta }}+\frac{-1}{2\beta }e^{\frac{-t^{2}}{4\beta }}+\frac{1}{2\beta }e^{\frac{-t^{2}}{4\beta }}\;\right]}{\frac{-3}{t^{4}}e^{\frac{-t^{2}}{4\beta }}-\frac{1}{2\beta t^{2}}e^{\frac{-t^{2}}{4\beta }}}],$$

whence, after algebraic manipulations, due to (15) we have

$$\underset{t\to \infty \;}{lim\;}t\phi_{2}\left(t,\beta \right)=e{\left(\frac{1}{3}\right)}^{\frac{3}{2}}\underset{t\to \infty \;}{lim\;}\left[\frac{\sqrt{\frac{\pi e}{2}}\;\;\frac{2}{t^{3}}erfc\left(\frac{t}{2\sqrt{\beta }}\right)}{\frac{3}{t^{4}}e^{\frac{-t^{2}}{4\beta }}+\frac{1}{2\beta t^{2}}e^{\frac{-t^{2}}{4\beta }}}\right]=e{\left(\frac{1}{3}\right)}^{\frac{3}{2}}\underset{t\to \infty \;}{lim\;}\left[\frac{\;\;\frac{2}{t^{3}}\phi_{0}\left(t,\beta \right)}{\frac{3}{t^{4}}+\frac{1}{2\beta t^{2}}}\right],$$

which, by (A9), finally yields

$$\underset{t\to \infty \;}{lim\;}t\phi_{2}\left(t,\beta \right)=e{\left(\frac{1}{3}\right)}^{\frac{3}{2}}\underset{t\to \infty \;}{lim\;}\left[\frac{\;\;\frac{2}{t^{3}}\phi_{0}\left(t,\beta \right)}{\frac{3}{t^{4}}+\frac{1}{2\beta t^{2}}}\right]=e{\left(\frac{1}{3}\right)}^{\frac{3}{2}}\underset{t\to \infty \;}{lim\;}\left[\frac{\;\;2\phi_{0}\left(t,\beta \right)}{\frac{3}{t}+\frac{1}{2\beta }t}\right]=0.$$

Thus, Property (18), for k=2, follows directly from (14), (A9) and (A11).

In the induction step, we assume that (18) and (19) hold for k and k−1, where k≥2. We prove that (18) and (19) hold also for k+1. By (14), in view of the induction hypothesis, in particular, due to (18) for k−1 and (19) for k, we immediately have

$$\underset{t\to \infty \;}{lim}\phi_{k+1}\left(t,\beta \right)=e{\left(\frac{k}{k+2}\right)}^{\frac{k+2}{2}}\left[\underset{t\to \infty \;}{lim}\phi_{k-1}\left(t,\beta \right)-\frac{1}{\sqrt{2\beta ek}\;}{\left(\frac{k+1}{k}\right)}^{\frac{k+1}{2}}\underset{t\to \infty \;}{lim}t\phi_{k}\left(t,\beta \right)\right]=0.$$

Keeping in mind (14), the product $t\phi_{k+1}\left(t,\beta \right)$ is

$$t\phi_{k+1}\left(t,\beta \right)=e\;{\left(\frac{k}{k+2}\right)}^{\frac{k+2}{2}}\left[t\phi_{k-1}\left(t,\beta \right)-\frac{1}{\sqrt{2\beta e}\;\sqrt{k}}{\left(\frac{k+1}{k}\right)}^{\frac{k+1}{2}}t^{2}\phi_{k}\left(t,\beta \right)\right],$$

whereby the induction hypothesis $t\phi_{k-1}\left(t,\beta \right)$ becomes zero, when t → ∞. Thus, it is only necessary to show that $t^{2}\phi_{k}\left(t,\beta \right)$ tend to zero as t→∞. Since both the numerator and denominator if the right fraction in

$$t^{2}\phi_{k}\left(t,\beta \right)=\frac{t^{2}}{\frac{1}{\phi_{k}\left(t,\beta \right)}},$$

tend to infinity as t→∞, applying the L’Hospital’s rule we obtain

$$\underset{t\to \infty \;}{lim}t^{2}\phi_{k}\left(t,\beta \right)=\underset{t\to \infty \;}{lim}\frac{t^{2}}{\frac{1}{\phi_{k}\left(t,\beta \right)}}=\underset{t\to \infty \;}{lim}\frac{2t}{\frac{-1}{{\left[\phi_{k}\left(t,\beta \right)\right]}^{2}}\;\frac{\partial \phi_{k}\left(t,\beta \right)}{\partial t}},$$

which, in view of (A1), can be expressed as

$$\underset{t\to \infty \;}{lim}t^{2}\phi_{k}\left(t,\beta \right)=\frac{2}{\sqrt{\frac{k+2}{2\beta e}}{\left(\frac{k+2}{k+1}\right)}^{\frac{k+1}{2}}}\underset{t\to \infty \;}{lim}\frac{t\phi_{k}\left(t,\beta \right)\phi_{k}\left(t,\beta \right)}{\;\phi_{k+1}\left(t,\beta \right)},$$

and next, including the formula (14), as follows

(A12) $$\underset{t\to \infty \;}{lim}t^{2}\phi_{k}\left(t,\beta \right)=4\beta e{\left(\frac{k}{k+2}\right)}^{\frac{k+2}{2}}\underset{t\to \infty \;}{lim}\left[\frac{\phi_{k-1}\left(t,\beta \right)\phi_{k}\left(t,\beta \right)}{\;\phi_{k+1}\left(t,\beta \right)}-\frac{1}{e}{\left(\frac{k+2}{k}\right)}^{\frac{k+2}{2}}\phi_{k}\left(t,\beta \right)\right],$$

where the second summand in the square brackets of the right-hand side tend to zero as t→∞, according to the induction hypothesis. Since by (14) the first summand can be expressed as a fraction

$$\frac{\phi_{k-1}\left(t,\beta \right)\phi_{k}\left(t,\beta \right)}{\;\phi_{k+1}\left(t,\beta \right)}=\frac{\frac{1}{e}{\left(\frac{k+2}{k}\right)}^{\frac{k+2}{2}}\phi_{k-1}\left(t,\beta \right)\phi_{k}\left(t,\beta \right)}{\phi_{k-1}\left(t,\beta \right)-\frac{1}{\sqrt{2\beta ek}\;}{\left(\frac{k+1}{k}\right)}^{\frac{k+1}{2}}t\phi_{k}\left(t,\beta \right)}=\frac{\frac{1}{e}{\left(\frac{k+2}{k}\right)}^{\frac{k+2}{2}}}{\frac{1}{\phi_{k}\left(t,\beta \right)}-\frac{1}{\sqrt{2\beta ek}\;}{\left(\frac{k+1}{k}\right)}^{\frac{k+1}{2}}\frac{t}{\phi_{k-1}\left(t,\beta \right)}},$$

which denominator tends to infinity as t→∞, also the first summand in the square brackets of the right hand side of (A12) become zero as t→∞. Thus,

$$\underset{t\to \infty \;}{lim}t^{2}\phi_{k}\left(t,\beta \right)=0,$$

i.e., (21) is proved, whence also (19) follows for k+1. This completes the proof. □

### Appendix A.3. Proof of Proposition 1

Since symmetric matrix $\Omega_{K,K}\left(\gamma \right)$ (33) is positive definite for any γ>0, the dual function $L_{D}\left(\lambda ,\gamma \right)$ (34) is strictly concave function of λ. By the following differential property [50]:

(A13) $$\frac{\partial }{\partial x}\left[A\left(x\right)B\left(x\right)\right]=\frac{\partial A\left(x\right)}{\partial x}B\left(x\right)+A\left(x\right)\frac{\partial B\left(x\right)}{\partial x},$$

which holds for arbitrary differentiable matrix functions A(x) and B(x), the first derivative of the dual function (34) with respect to γ is as follows

(A14) $$\frac{\partial }{\partial \gamma }L_{D}\left(\lambda ,\gamma \right)=-\kappa^{2}-{\left(\Phi_{N,K}^{T}{\bar{G}}_{N}+\frac{1}{2}\lambda \right)}^{T}\frac{\partial \Omega_{K,K}\left(\gamma \right)}{\partial \gamma }\left(\Phi_{N,K}^{T}{\bar{G}}_{N}+\frac{1}{2}\lambda \right),$$

while the second derivative is

(A15) $$\frac{\partial^{2}}{\partial \gamma^{2}}L_{D}\left(\lambda ,\gamma \right)=-{\left(\Phi_{N,K}^{T}{\bar{G}}_{N}+\frac{1}{2}\lambda \right)}^{T}\frac{\partial^{2}\Omega_{K,K}\left(\gamma \right)}{\partial \gamma^{2}}\left(\Phi_{N,K}^{T}{\bar{G}}_{N}+\frac{1}{2}\lambda \right).$$

By the next differential property [50]:

(A16) $$\frac{\partial }{\partial x}\left[A{\left(x\right)}^{-1}\right]=-A{\left(x\right)}^{-1}\frac{\partial A\left(x\right)}{\partial x}A{\left(x\right)}^{-1},$$

which holds for arbitrary differentiable and invertible matrix function A(x), for $\Omega_{K,K}\left(\gamma \right)$ (33) we have

(A17) $$\frac{\partial }{\partial \gamma }\Omega_{K,K}\left(\gamma \right)=\frac{\partial }{\partial \gamma }{\left(\Phi_{N,K}^{T}\Phi_{N,K}+\gamma I_{K,K}\right)}^{-1}=-{\left(\Phi_{N,K}^{T}\Phi_{N,K}+\gamma I_{K,K}\right)}^{-2}=-\Omega_{K,K}^{2}\left(\gamma \right).$$

Whence, keeping in mind (33), (A13), (A16) and (A17), we obtain

(A18) $$\frac{\partial^{2}\Omega_{K,K}\left(\gamma \right)}{\partial \gamma^{2}}=-\frac{\partial }{\partial \gamma }\Omega_{K,K}^{2}\left(\gamma \right)=2{\left(\Phi_{N,K}^{T}\Phi_{N,K}+\gamma I_{K,K}\right)}^{-3}=2\Omega_{K,K}^{3}(\gamma ).$$

Combining (A14) and (A17) yields

(A19) $$\frac{\partial }{\partial \gamma }L_{D}\left(\lambda ,\gamma \right)=-\kappa^{2}+{\left(\Phi_{N,K}^{T}{\bar{G}}_{N}+\frac{1}{2}\lambda \right)}^{T}\Omega_{K,K}^{2}\left(\gamma \right)\left(\Phi_{N,K}^{T}{\bar{G}}_{N}+\frac{1}{2}\lambda \right),$$

while, by (A18) and (A15), the second derivative is as follows

$$\frac{\partial^{2}}{\partial \gamma^{2}}L_{D}\left(\lambda ,\gamma \right)=-2{\left(\Phi_{N,K}^{T}{\bar{G}}_{N}+\frac{1}{2}\lambda \right)}^{T}\Omega_{K,K}^{3}\left(\gamma \right)\left(\Phi_{N,K}^{T}{\bar{G}}_{N}+\frac{1}{2}\lambda \right).$$

Thus, $L_{D}\left(\lambda ,\gamma \right)$ is strictly concave function of the multiplier γ, too.

Differentiating $\frac{\partial }{\partial \gamma }L_{D}\left(\lambda ,\gamma \right)$ (A19) with respect to λ and taking into account the quadratic form of λ given by (34), it is easy to check that the Hessian matrix of the dual function takes the form:

$$ℍ\left(\lambda ,\gamma \right)=\;\left[\begin{array}{cc} -\frac{1}{2}\Omega_{K,K}\left(\gamma \right) & \Omega_{K,K}^{2}\left(\gamma \right)\left(\Phi_{N,K}^{T}{\bar{G}}_{N}+\frac{1}{2}\lambda \right)\\ {\left(\Phi_{N,K}^{T}{\bar{G}}_{N}+\frac{1}{2}\lambda \right)}^{T}\Omega_{K,K}^{2}\left(\gamma \right) & -2{\left(\Phi_{N,K}^{T}{\bar{G}}_{N}+\frac{1}{2}\lambda \right)}^{T}\Omega_{K,K}^{3}\left(\gamma \right)\left(\Phi_{N,K}^{T}{\bar{G}}_{N}+\frac{1}{2}\lambda \right) \end{array}\right].$$

Since $\Omega_{K,K}\left(\gamma \right)$ (33) is positive definite, the Hessian ℍ(λ,γ) is negative definite for an arbitrary $\lambda \geq 0_{K}$, on the basis of the known result concerning the definiteness of block matrices [55] (Twierdzenia I_b’’_). Thus, dual function $L_{D}\left(\lambda ,\gamma \right)$ is strictly concave function of both arguments (λ,γ). Theorem is proved. □

### Appendix A.4. Proof of Theorem 4

In the proof, the following Rayleigh-Ritz inequalities [56] (Lemma I) which hold for any $x\epsilon {ℛ}^{m}$ and any symmetric matrix $X=X^{T}\epsilon {ℛ}^{m,m}$:

(A20) $$\lambda_{min}\left(X\right)x^{T}x\leq x^{T}Xx\leq \lambda_{max}\left(X\right)x^{T}x,$$

is used, where $\lambda_{min}\left(X\right)$ and $\lambda_{max}\left(X\right)$ are minimal and maximal eigenvalues of X.

Since for any γ>0 matrix $\Omega_{K,K}\left(\gamma \right)$ (33) is positive definite, using the left inequality from (A20) to estimate the last term of the right-hand side of (34), we obtain the following inequality

(A21) $$L_{D}\left(\lambda ,\gamma \right)\leq {\bar{G}}_{N}^{T}{\bar{G}}_{N}-\gamma \kappa^{2}-\lambda_{min}\left[\Omega_{K,K}\left(\gamma \right)\right]{\left(Y_{K}+\frac{1}{2}\lambda \right)}^{T}\left(Y_{K}+\frac{1}{2}\lambda \right),$$

where $\lambda_{min}\left[\Omega_{K,K}\left(\gamma \right)\right]>0$ is minimal eigenvalue of $\Omega_{K,K}\left(\gamma \right)$ and vector $Y_{K}$ is defined by (40). By (36) and (37), we have $\lambda_{min}\left[\Omega_{K,K}\left(\gamma \right)\right]=1/\left(\sigma_{1}^{2}+\gamma \right)$, whenever γ>0, whence inequality (A21) can be rewritten as

(A22) $$L_{D}\left(\lambda ,\gamma \right)\leq {\bar{G}}_{N}^{T}{\bar{G}}_{N}-\gamma \kappa^{2}-\frac{1}{\left(\sigma_{1}^{2}+\gamma \right)}{\left(Y_{K}+\frac{1}{2}\lambda \right)}^{T}\left(Y_{K}+\frac{1}{2}\lambda \right)=L_{Dupp}\left(\lambda ,\gamma \right).$$

Let $\left(\lambda_{0},\gamma_{0}\right)$ be a pair of arbitrary allowable Lagrange multipliers, i.e., $\lambda_{0}\geq 0_{K}$ and $\gamma_{0}\geq 0$.

The upper bound $L_{Dupp}\left(\lambda ,\gamma \right)$ of $L_{D}\left(\lambda ,\gamma \right)$, defined by the right-hand side equation in (A22), is strictly concave function of γ, which for

(A23) $$\gamma_{max}\left(\lambda \right)=\frac{\sqrt{{\left(Y_{K}+\frac{1}{2}\lambda \right)}^{T}\left(Y_{K}+\frac{1}{2}\lambda \right)}}{\kappa }-\sigma_{1}^{2}$$

takes maximum, with respect to γ, given by

(A24) $$L_{Dupp}\left(\lambda ,\gamma_{max}\left(\lambda \right)\right)={\bar{G}}_{N}^{T}{\bar{G}}_{N}+\sigma_{1}^{2}\kappa^{2}-2\kappa \sqrt{{\left(Y_{K}+\frac{1}{2}\lambda \right)}^{T}\left(Y_{K}+\frac{1}{2}\lambda \right)}.$$

For the non-negative definite basis functions $\varphi_{k}\left(t,\alpha \right)$ (7) or $\phi_{k}\left(t,\beta \right)$ (14)–(16), the inequality $Y_{K}>0_{K}$ holds, since the relaxation modulus measurements are non-negative. Therefore, all elements of the vector $Y_{K}$ are positive. Thus, in view of the assumption (39), $\gamma_{max}\left(\lambda \right)$ given by (A23) is positive for any $\lambda \geq 0_{K}$.

There exists a positive constant

(A25) $$M_{0}=\left(\gamma_{0}+\sigma_{1}^{2}\right)\kappa +\frac{1}{\kappa }{\left(Y_{K}+\frac{1}{2}\lambda_{0}\right)}^{T}\Omega_{K,K}\left(\gamma_{0}\right)\left(Y_{K}+\frac{1}{2}\lambda_{0}\right),$$

such that for any $\lambda \geq 0_{K}$, inequality ‖**λ**‖_2_ > *M*_0_ implies

(A26) $$L\left(\lambda ,\gamma \right)\leq L_{Dupp}\left(\lambda ,\gamma \right)\leq L_{Dupp}\left(\lambda ,\gamma_{max}\left(\lambda \right)\right)<L_{D}\left(\lambda_{0},\gamma_{0}\right).$$

for any γ>0. Indeed, if $\;\lambda_{2}>M_{0}$, then having in mind non-negativity of the vectors $Y_{K}$ and λ, by (A25) we have

$${\left(Y_{K}+\frac{1}{2}\lambda \right)}^{T}\left(Y_{K}+\frac{1}{2}\lambda \right)\geq \frac{1}{4}\|\lambda {\|}_{2}^{2}>{\left[\left(\gamma_{0}+\sigma_{1}^{2}\right)\frac{\kappa }{2}+\frac{1}{2\kappa }{\left(Y_{K}+\;\frac{1}{2}\lambda_{0}\right)}^{T}\Omega_{K,K}\left(\gamma_{0}\right)\left(Y_{K}+\frac{1}{2}\lambda_{0}\right)\right]}^{2},$$

whence the following inequality follows from extreme, left and right, expressions

$$2\kappa \sqrt{{\left(Y_{K}+\frac{1}{2}\lambda \right)}^{T}\left(Y_{K}+\frac{1}{2}\lambda \right)}>\left(\gamma_{0}+\sigma_{1}^{2}\right)\kappa^{2}+{\left(Y_{K}+\frac{1}{2}\lambda_{0}\right)}^{T}\Omega_{K,K}\left(\gamma_{0}\right)\left(Y_{K}+\frac{1}{2}\lambda_{0}\right).$$

Thus, by (A24) and (34), the next estimation holds

$$L_{Dupp}\left(\lambda ,\gamma_{max}\left(\lambda \right)\right)<L_{D}\left(\lambda_{0},\gamma_{0}\right),$$

and, due to (A22), implies (A26) for an arbitrary γ>0, which, in view of the left inequality in (A22), means that the maximization of the dual function $L_{D}\left(\lambda ,\gamma \right)$ with respect to λ can be restricted to $\lambda \geq 0_{K}$ such that $\|\lambda {\|}_{2}\leq M_{0}$.

Similarly, for an arbitrary $\gamma >\gamma_{2}$, where

$$\gamma_{2}=\gamma_{0}+\frac{1}{\kappa^{2}}{\left(Y_{K}+\frac{1}{2}\lambda_{0}\right)}^{T}\Omega_{K,K}\left(\gamma_{0}\right)\left(Y_{K}+\frac{1}{2}\lambda_{0}\right),$$

which yields that

$$\gamma \kappa^{2}>\gamma_{2}\kappa^{2}=\gamma_{0}\kappa^{2}+{\left(Y_{K}+\frac{1}{2}\lambda_{0}\right)}^{T}\Omega_{K,K}\left(\gamma_{0}\right)\left(Y_{K}+\frac{1}{2}\lambda_{0}\right),$$

by (34), the next inequality folds

$$L_{D}\left(\lambda ,\gamma \right)<L_{D}\left(\lambda_{0},\gamma_{0}\right)$$

for any $\lambda \geq 0_{K}$. Thus, maximization of the dual function with respect to γ can be restricted to the subset $0\leq \gamma \leq \gamma_{2}$, independently of the value of $\lambda \geq 0_{K}$.

Combining the above, the maximization in the dual optimization task (30) can be constrained to compact subset of the pairs (λ,γ) in the Cartesian product ${ℛ}_{+}^{K}\times {ℛ}_{+}$ defined by inequalities

$${\left\|\lambda \right\|}_{2}\leq M_{0}\;\;\;\mathrm{and}\;0\leq \gamma \leq \gamma_{2},$$

here, ${ℛ}_{+}$ denotes the set of non-negative real numbers. Thus, by view of the Weierstrass theorem concerning the extreme of continuous function on the compact set [52], the existence of the solution to the dual problem is immediately concluded. To demonstrate that the optimal price $\hat{\gamma }>0$, we show that for any $\lambda \geq 0_{K}$ inequality (39) imply

$$\underset{\gamma \to 0^{+}}{\lim}\frac{\partial }{\partial \gamma }L_{D}\left(\lambda ,\gamma \right)>0,$$

i.e., the dual function grows, as a function of γ, in the near right neighborhood of the zero. To show the above, note that by (A19) and the left inequality in (A20), having in mind that due to (36) and (37) $\lambda_{min}\left[\Omega_{K,K}^{2}\left(\gamma \right)\right]=1/{\left(\sigma_{1}^{2}+\gamma \right)}^{2}$, we have

(A27) $$\frac{\partial }{\partial \gamma }L_{D}\left(\lambda ,\gamma \right)\geq -\kappa^{2}+\frac{1}{{\left(\sigma_{1}^{2}+\gamma \right)}^{2}}{\left(Y_{K}+\frac{1}{2}\lambda \right)}^{T}\left(Y_{K}+\frac{1}{2}\lambda \right).$$

The assumption (39) imply

$$-\kappa^{2}+\frac{1}{\sigma_{1}^{4}}Y_{K}^{T}Y_{K}>0.$$

Thus, there exists $\gamma_{1}>0$, for example

$$\gamma_{1}=\frac{\sqrt{Y_{K}^{T}Y_{K}}}{\kappa }-\sigma_{1}^{2},$$

such that for any $\lambda \geq 0_{K}$, having in mind that also $Y_{K}\geq 0_{K}$, by (A27), the inequality $\frac{\partial }{\partial \gamma }L_{D}\left(\lambda ,\gamma \right)>0$ holds for any $0<\gamma <\gamma_{1}$, i.e., the dual function increases with growing γ in the near neighborhood of γ=0. Therefore, the optimal price $\hat{\gamma }\geq \gamma_{1}>0$, which completes the proof. □

### Appendix A.5. Derivation of the Formula (47)

The function ${\bar{L}}_{D}\left(\lambda \right)$ (46) can be expressed as

(A28) $${\bar{L}}_{D}\left(\lambda \right)={\bar{G}}_{N}^{T}{\bar{G}}_{N}-\kappa^{2}\bar{\gamma }\left(\lambda \right)-\Gamma \left(\lambda \right),$$

where the last summand, by (46) and (40), is as follows

(A29) $$\Gamma \left(\lambda \right)={\left(Y_{K}+\frac{1}{2}\lambda \right)}^{T}\Omega_{K,K}\left(\bar{\gamma }\left(\lambda \right)\right)\left(Y_{K}+\frac{1}{2}\lambda \right).$$

Let $\lambda_{k}$ be the k-th element of the vector λ, k=1,…,K. Thus, (A28) yields

(A30) $$\frac{\partial }{\partial \lambda_{k}}{\bar{L}}_{D}\left(\lambda \right)=-\kappa^{2}\frac{\partial \bar{\gamma }\left(\lambda \right)}{\partial \lambda_{k}}-\frac{\partial }{\partial \lambda_{k}}\Gamma \left(\lambda \right).$$

By (A29) and (A13), we have

(A31) $$\frac{\partial }{\partial \lambda_{k}}\Gamma \left(\lambda \right)={\left(Y_{K}+\frac{1}{2}\lambda \right)}^{T}\Omega_{K,K}\left(\bar{\gamma }\left(\lambda \right)\right)J_{K}^{k}+{\left(Y_{K}+\frac{1}{2}\lambda \right)}^{T}\frac{\partial \Omega_{K,K}\left(\bar{\gamma }\left(\lambda \right)\right)}{\partial \lambda_{k}}\left(Y_{K}+\frac{1}{2}\lambda \right),$$

where $J_{K}^{k}$ is K-dimensional vector, which k element is equal one, while the other elements are zero. Since

$$\frac{\partial }{\partial \lambda_{k}}\left(\Phi_{N,K}^{T}\Phi_{N,K}+\bar{\gamma }\left(\lambda \right)I_{K,K}\right)=\frac{\partial \bar{\gamma }\left(\lambda \right)}{\partial \lambda_{k}}I_{K,K},$$

due to (33) and (A16), we obtain

(A32) $$\frac{\partial \Omega_{K,K}\left(\bar{\gamma }\left(\lambda \right)\right)}{\partial \lambda_{k}}=-\frac{\partial \bar{\gamma }\left(\lambda \right)}{\partial \lambda_{k}}{\left(\Phi_{N,K}^{T}\Phi_{N,K}+\bar{\gamma }\left(\lambda \right)I_{K,K}\right)}^{-2}=-\frac{\partial \bar{\gamma }\left(\lambda \right)}{\partial \lambda_{k}}\Omega_{K,K}^{2}\left(\bar{\gamma }\left(\lambda \right)\right).$$

Combining (A30), (A31) and (A32) yields

$$\frac{\partial {\bar{L}}_{D}\left(\lambda \right)}{\partial \lambda_{k}}=-\kappa^{2}\frac{\partial \bar{\gamma }\left(\lambda \right)}{\partial \lambda_{k}}-{\left(Y_{K}+\frac{1}{2}\lambda \right)}^{T}\Omega_{K,K}\left(\bar{\gamma }\left(\lambda \right)\right)J_{K}^{k}+\frac{\partial \bar{\gamma }\left(\lambda \right)}{\partial \lambda_{k}}{\left(Y_{K}+\frac{1}{2}\lambda \right)}^{T}\Omega_{K,K}^{2}\left(\bar{\gamma }\left(\lambda \right)\right)\left(Y_{K}+\frac{1}{2}\lambda \right),$$

whence, in view of (43) and (40), we obtain

$$\frac{\partial }{\partial \lambda_{k}}{\bar{L}}_{D}\left(\lambda \right)=-{\left(Y_{K}+\frac{1}{2}\lambda \right)}^{T}\Omega_{K,K}\left(\bar{\gamma }\left(\lambda \right)\right)J_{K}^{k}=-{\left(J_{K}^{k}\right)}^{T}\Omega_{K,K}\left(\bar{\gamma }\left(\lambda \right)\right)\left(Y_{K}+\frac{1}{2}\lambda \right),$$

Therefore, having in mind (33) and (40), the formula (47) describing the gradient $\frac{\partial }{\partial \lambda }{\bar{L}}_{D}\left(\lambda \right)$ immediately results. □

### Appendix A.6. Proof of Proposition 2

By (10), for any factor β and any vector $g_{K}$, we have

(A33) $$\|{H_{K}(v,\beta )\|}_{2}^{2}=\int_{0}^{\infty }{\left[\sum_{k=0}^{K-1}g_{k}h_{k}\left(v,\beta \right)\right]}^{2}dv,$$

which can be rewritten as

$$\|{H_{K}(v,\beta )\|}_{2}^{2}=\sum_{k=0}^{K-1}\sum_{j=0}^{K-1}g_{k}g_{j}\vartheta_{kj}\left(\beta \right),$$

where the functions

(A34) $$\vartheta_{kj}\left(\beta \right)=\int_{0}^{\infty }h_{k}\left(v,\beta \right)h_{j}\left(v,\beta \right)dv$$

are such that $\vartheta_{kj}\left(\beta \right)=\vartheta_{jk}\left(\beta \right)$ for k,j=0,1,…K−1.

By (11) and (A34) we have

$$\vartheta_{kj}\left(\beta \right)={\left(\frac{2\beta e}{k+1}\right)}^{\frac{k+1}{2}}{\left(\frac{2\beta e}{j+1}\right)}^{\frac{j+1}{2}}\int_{0}^{\infty }v^{k+j+2}e^{-2\beta v^{2}}dv,$$

whence, having in mind (11) and (13), the next equality results

$$\vartheta_{kj}\left(\beta \right)=\frac{\sqrt{k+j+3}}{\sqrt{2\beta e}}\frac{{\left(\frac{k+j+3}{k+1}\right)}^{\frac{k+1}{2}}{\left(\frac{k+j+3}{j+1}\right)}^{\frac{j+1}{2}}}{2^{\frac{k+j+3}{2}}}\phi_{k+j+2}\left(0,2\beta \right)=\frac{1}{\sqrt{2\beta e}}\theta_{kj}\left(\beta \right).$$

Thus, $\vartheta_{kj}\left(\beta \right)$ and $\theta_{kj}\left(\beta \right)$ (56) are uniquely determined by the basis functions $\phi_{k}\left(t,\beta \right)$ for t=0. By the recurrent formula (14), for t=0, we have

$$\phi_{k+1}\left(0,2\beta \right)=e{\left(\frac{k}{k+2}\right)}^{\frac{k+2}{2}}\phi_{k-1}\left(0,2\beta \right).$$

Since erfc(x)=1, by (15), we have $\phi_{0}\left(0,2\beta \right)=\sqrt{\pi e/2}\;$, and (16) yields $\phi_{1}\left(0,2\beta \right)=e/2\;$. Thus, it is easy to check, by algebraic manipulations, that for even indices formula (57) holds, while for odd indices (58) is satisfied, where k=1,2,….

According to (A33) and (55), the quadratic form $g_{K}^{T}\Theta g_{K}$ is expressed as

$$g_{K}^{T}\Theta g_{K}=\sqrt{2\beta e}\;\int_{0}^{\infty }{\left[\sum_{k=0}^{K-1}g_{k}h_{k}\left(v,\beta \right)\right]}^{2}dv.$$

Thus, $g_{K}^{T}\Theta g_{K}\geq 0$ for an arbitrary vector $g_{K}$, and $g_{K}^{T}\Theta g_{K}=0$, if and only if $\sum_{k=0}^{K-1}g_{k}h_{k}\left(v,\beta \right)=0$ for almost all v>0. Since the basis functions $h_{k}\left(v,\beta \right)$ are independent, the last equality holds, if and only if $g_{k}=0$ for all k=0,1,…,K−1, i.e., only if the vector $g_{K}=0$, which yields the positive definiteness of Θ. Proposition is proved. □

## Appendix B

**Table A1:** Ranges of the applicability of the models (10) and (12) for various time-scale parameters for K=6,…11.

Time-Scale Factor β $\left[s^{2}\right]$	Range ^1^ of Relaxation Frequencies $v_{app}\left(\beta \right)$ $\left[s^{-1}\right]$	Range^1^ of Times $t_{app}\left(\beta \right)$ [s]	Range ^1^ of Relaxation Frequencies $v_{app}\left(\beta \right)$ $\left[s^{-1}\right]$	Range ^1^ of Times $t_{app}\left(\beta \right)$ [s]
	K=6	K=6	K=7	K=7
0.0000001	11,227.86	0.006284	11,632.60	0.006284
0.000001	3550.95	0.01987	3678.97	0.01987
0.00001	1.122.75	0.0628	1163.50	0.0628
0.0001	3.55.20	0.199	368.10	0.199
0.001	1.12.50	0.628	116.40	0.628
0.01	35.53	1.987	36.86	1.987
0.1	11.305	6.284	11.685	6.284
1	3.60	19.872	3.69	19.872
10	1.125	62.870	1.17	62.870
100	0.36	198.57	0.37	198.57
β $\left[s^{2}\right]$	K=8	K=8	K=9	K=9
0.0000001	12,012.84	0.006284	12,372.50	0.006284
0.000001	3798.96	0.01987	3912.97	0.01987
0.00001	1201.50	0.0628	1237.50	0.0628
0.0001	380.06	0.199	391.53	0.199
0.001	120.30	0.628	123.90	0.628
0.01	38.18	1.987	39.33	1.987
0.1	12.065	6.284	12.445	6.284
1	3.825	19.872	3.915	19.872
10	1.206	62.870	1.242	62.870
100	0.38	198.57	0.395	198.57
β $\left[s^{2}\right]$	K=10	K=10	K=11	K=11
0.0000001	12,714.52	0.006284	13,041.84	0.006284
0.000001	4020.80	0.01987	4124.535	0.01987
0.00001	1271.60	0.0628	1304.50	0.0628
0.0001	402.20	0.199	412.61	0.199
0.001	127.20	0.628	130.50	0.628
0.01	40.22	1.987	41.40	1.987
0.1	12.73	6.284	13.11	6.284
1	4.05	19.872	4.14	19.872
10	1.278	62.870	1.3050	62.870
100	0.405	198.57	0.4150	198.57

^1^ The upper bounds $t_{app}\left(\beta \right)$ (23) and $v_{app}\left(\beta \right)$ (24) of the applicability intervals $\left[0,t_{app}\left(\beta \right)\right]$ and $\left[0,v_{app}\left(\beta \right)\right]$ are given.

**Table A2:** Optimal non-negative parameters ${\hat{g}}_{K}$ of the relaxation spectrum models from Example 1 for K=3,…,12 numbers of model components K=3,…,12; the elements of the vectors are expressed in [Pa].

**Non-Negative Optimal Model Parameters** ${\hat{g}}_{K}$									
K=3	K=4	K=5	K=6	K=7	K=8	K=9	K=10	K=11	K=12
0.51610	0.60302	0.52025	0.43015	0.33212	0.23311	0.13764	0.04694	0	0
0.47285	0.03345	0.24236	0.39697	0.54005	0.67254	0.79475	0.89649	0.96617	0.98616
0	0	0	0	0	0	0	0	0	0.01056
	0.99558	0	0	0	0	0	0	0	0
		0.98433	0	0	0	0	0	0	0
			1.01872	0	0	0	0	0	0
				1.03876	0	0	0	0	0
					1.04386	0	0	0	0
						1.03356	0	0	0
							1.05751	0	0
								1.07919	0
									1.14125

**Table A3:** Optimal non-constrained ${\tilde{g}}_{K}$ and non-negative parameters ${\hat{g}}_{K}$ of the relaxation spectrum models from Example 2 for selected numbers of model components *K*; the elements of both vectors are expressed in [Pa].

Non-Constrained Optimal Model Parameters ${\tilde{g}}_{K}$									
K=6	K=8	K=10	K=12	K=14	K=15	K=16	K=18	K=20	K=21
0.69589	1.23437	2.48294	3.04854	0.63923	0.54198	0.57961	0.478723	0.40709	0.36393
−1.77431	−3.18711	−2.69711	5.24032	0.03965	0.33562	0.21153	0.16921	0.21656	0.11202
1.91327	1.39411	−3.13256	−12.91242	−3.423× 10^−5^	−0.05921	−0.03719	0.11940	0.12699	0.14692
0.78759	1.87886	3.41395	1.72539	0.076367	0.38651	0.35181	0.11929	0.16832	0.00767
0.08083	0.40102	1.38949	2.44539	−0.43247	−0.66386	−0.60506	−0.22342	−0.25073	0.13480
0.00327	0.05369	0.42147	1.85665	0.201602	−0.64325	−0.45072	0.10231	0.08018	0.08627
	0.00411	0.08935	0.91252	0.70569	0.29752	0.39431	0.12578	−0.04074	0.082852
	1.37 × 10^−4^	0.01230	0.30148	0.60727	0.91472	0.84376	−0.00747	−0.12579	0.19501
		9.822 × 10^−4^	0.06676	0.29290	0.77923	0.66133	0.186577	0.17558	0.101948
		3.448 × 10^−5^	9.537 × 10^−3^	0.09022	0.38086	0.30802	0.47089	0.18924	0.15143
			7.963 × 10^−4^	0.018247	0.12026	0.09394	0.48281	0.06758	0.22989
			2.959 × 10^−5^	2.362 × 10^−3^	0.02511	0.01909	0.29154	0.28282	0.14736
				1.785 × 10^−4^	3.372 × 10^−3^	2.515 × 10^−3^	0.11590	0.54995	0.14569
				6.015 × 10^−6^	2.652 × 10^−4^	1.962 × 10^−4^	0.03149	0.51033	0.25043
					9.326 × 10^−6^	7.093 × 10^−6^	5.837 × 10^−3^	0.28295	0.26079
						2.279 × 10^−8^	7.103 × 10^−4^	0.10182	0.16058
							5.137 × 10^−5^	0.02424	0.06268
							1.679 × 10^−6^	3.712 × 10^−3^	0.01589
								3.333 × 10^−4^	2.557 × 10^−3^
								1.339 × 10^−5^	2.388 × 10^−4^
									9.906 × 10^−6^
**Non−negative optimal model parameters** ${\hat{g}}_{K}$									
K=6	K=8	K=10	K=12	K=14	K=15	K=16	K=18	K=20	K=21
0.37194	0.10317	0.37144	0.50501	0	0	0.3370	0.516089	0.375427	0.36393
0	0	0	0	8.65 × 10^−4^	1.05 × 10^−5^	0.24060	0	0	0.11202
1.16788	1.64821	0.72971	9.308 × 10^−7^	1.36631	1.5380	0	0	1.33 × 10^−10^	0.14692
0.15572	0	0.53285	0	0	0.18528	0.01840	0	0	0.00767
3.601 × 10^−3^	0	0.10922	0	0.36131	0.02458	0	0	0	0.13480
0	0.01015	1.051 × 10−^8^	0.74577	0.18804	0	0	0.339032	0.367328	0.08627
	1.956 × 10^−3^	0	0.700892	0.03469	2.119 × 10^−3^	0.41948	0.200519	0.294	0.082852
	1.069 × 10^−4^	6.772 × 10^−4^	0.28695	2.257 × 10^−3^	7.336 × 10−^4^	0.84380	0	0	0.19501
		8.569 × 10^−5^	0.06676	0	6.418 × 10^−5^	0.66133	0.186117	0.148038	0.101948
		9.67 × 10^−7^	9.537 × 10^−3^	4.0 × 10^−5^	2.07 × 10−^6^	0.30802	0.470796	0.160911	0.15143
			7.963 × 10^−4^	1.717 × 10^−5^	2.756 × 10−^7^	0.09394	0.482808	0.061062	0.22989
			2.960 × 10^−5^	3.74 × 10^−6^	0	0.01909	0.291539	0.282294	0.14736
				5.60 × 10^−7^	0	2.515 × 10^−3^	0.115905	0.54995	0.14569
				4.0 × 10^−8^	4.0 × 10^−10^	1.962 × 10^−4^	0.031492	0.510332	0.25043
					8.0 × 10^−11^	7.090 × 10^−6^	5.838 × 10^−3^	0.282955	0.26079
						2.280 × 10^−8^	7.10 × 10^−4^	0.101824	0.16058
							5.14 × 10^−5^	0.024238	0.06268
							1.68 × 10^−6^	3.712 × 10^−3^	0.01589
								3.33 × 10^−4^	2.557 × 10^−3^
								1.34 × 10^−5^	2.388 × 10^−4^
									9.906 × 10^−6^

**Table A4:** Optimal parameters ${\tilde{g}}_{K}$ and ${\hat{g}}_{K}$ of the best models ${\tilde{H}}_{K}\left(v,\beta \right)$ (62) and ${\hat{H}}_{K}\left(v,\beta \right)$ (54) of the relaxation spectrum from Example 3 determined without and with the non-negativity constraints, respectively, for time scale factors $\beta =\beta_{opt}$ given in Table 5; the elements of the vectors ${\hat{g}}_{K}$ are expressed in [Pa].

Optimal Model Parameters ${\tilde{g}}_{K}$ Determined without Non-Negativity Constraint					
K=3	K=4	K=5	K=6	K=7	K=8
−0.31279	−0.33746	−0.33855	−0.3194927	−0.33996837	−0.592754
0.15877	−0.03316	−0.19956	−0.3546004	−0.2678777	1.026829
1.94169	2.15237	2.2879722	2.3595877	2.34997477	2.10646
	0.01423	0.0474225	0.1037881	5.89358 × 10^−2^	−0.55997
		2.9359 × 10^−3^	0.01345137	9.9667 × 10^−4^	−0.19311
			7.7147 × 10^−4^	−1.09287 × 10^−3^	−4.00838 × 10^−2^
				−1.1487 × 10^−4^	−4.5905 × 10^−3^
					−2.229 × 10^−4^
**Optimal model parameters** ${\hat{g}}_{K}$ **determined with non−negativity constraint**					
K=3	K=4	K=5	K=6	K=7	K=8
0	0	0	0	0	0
1.5606281	0	0	0	0	0.18757
0	1.38536	1.24139	1.10756	1.0669564	1.19620
	0.22688	0.42096	0.502009	0.4743012	0.310804
		0.06093	0.168412	0.2304561	0.047481
			0.017565	0.0497706	0
				3.928854 × 10^−3^	2.9752 × 10^−4^
					1.11681 × 10^−4^
